# Gold Nanoturf‐Mediated Wireless Photothermal Upregulation of Human Adipose‐Derived Stem Cell Spheroids for Synergistic Skin‐Wound Closure

**DOI:** 10.1002/advs.202515490

**Published:** 2025-10-14

**Authors:** Jong Uk Kim, Jiyu Hyun, Gyan Raj Koirala, Jiwon Kim, Sung‐Won Kim, Gwang‐Bum Im, Yeong Hwan Kim, Young Gil Kim, Dong‐Hyun Lee, Hyun Su Park, Young‐Ju Jang, Young Jin Jo, Chanho Jeong, Arpan Koirala, Janghoon Joo, Sang Min Won, Suk Ho Bhang, Tae‐il Kim

**Affiliations:** ^1^ School of Chemical Engineering Sungkyunkwan University (SKKU) Suwon 16419 Republic of Korea; ^2^ Biomedical Institute for Convergence at SKKU (BICS) Sungkyunkwan University (SKKU) Suwon 16419 Republic of Korea; ^3^ Department of Computer Science and Engineering Kathmandu University Dhulikhel Kavre P.O. Box 6250 Nepal; ^4^ Department of Electrical and Computer Engineering Sungkyunkwan University (SKKU) Suwon 16419 Republic of Korea

**Keywords:** gold nanoturf structure, stem cell therapy, wireless photo‐biomodulation, wound healing

## Abstract

3D stem cell aggregates at the sub‐millimeter feature, known as spheroids, have garnered significant interest for their capability to effectively heal and close wound areas. Nevertheless, further efforts to unlock their maximal therapeutic potential and to address engineering challenges are required for medical/clinical translation. Herein, a wireless skin wound care platform designed for multimodal therapy, combining photothermal, stem cell, and light (PTSCL) effects to enhance therapeutic efficacy, is presented. The synergy is achieved by integrating a wireless LED module that irradiates visible light (630 nm‐wavelength) and human adipose‐derived stem cells (hADSCs) spheroid‐laden polydimethylsiloxane (PDMS) patch embedded with a light‐to‐heat converting membrane (i.e., Au‐coated nanoturf). This wireless platform primarily enables photothermal stimulation at the wound site, facilitating hADSC spheroid formation and upregulation via heat shock protein‐mediated mechanism, and effective delivery of spheroids from the patch. Additionally, the photothermal approach, comprising mild hyperthermia and light stimulation, independently utilized to promote wound healing, exhibits a synergistic therapeutic effect. Collected data from visual/computational analysis, in vitro assays, and in vivo experiments in a mouse wound model (2 cm‐by‐2 cm) provide evidence for the synergistic effect of the proposed PTSCL treatment and suggest its potential to be implemented for tissue regeneration for severe wound patients.

## Graphical abstract

A wireless skin‐wound care platform for photothermal‐stem cell‐light (PTSCL) therapy, introduced here, leverages a fully wireless and battery‐free optoelectronic module, gold nanoturf‐mediated photothermal interface, and human adipose‐derived stem cell (hADSC) spheroids to synergistically enhance wound healing. Besides accelerated skin wound closure, the proposed system shows efficacy regarding cell delivery, skin maturation, and angiogenesis.

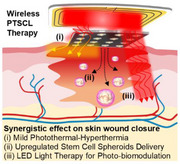

## 1 Introduction

The skin, the outermost layer of the human defense system, acts as a physical barrier against the direct invasion of harmful pathogens, including viruses and bacteria.^[^ ^1^ ^]^ However, such skin integrity is prone to being compromised by unexpected accidents, injuries, surgeries, or complications, which can potentially lead to severe chronic wounds.^[^ ^2^ ^]^ Due to the risk of infection exposure during the wound healing period, emerging wound healing therapies such as hydrogels, biodegradable polymers, cell delivery, and physical stimulation (e.g., electrical, thermal, light stimulation) over the wound area have been developed to accelerate wound closure,^[^ ^3^, ^4^, ^5^, ^6^, ^7^, ^8^ ^]^ which is the foremost goal of skin wound care. Compared with other approaches, stem cell therapy (e.g., mesenchymal stem cells (MSCs)) is captivated to tissue regeneration for wound care because of their remarkable therapeutic efficacy derived from the secretion of a variety of growth factors that assist immunomodulation, angiogenesis in the wound area, as well as recruiting cells for wound recovery.^[^ ^9^ ^]^ Additionally, previous studies revealed that external physical modulation, such as cytokine treatment, light irradiation, electrical stimulation, and mild hyperthermia^[^ ^10^, ^11^, ^12^ ^]^ could upregulate stem cells for enhanced therapeutic results.

Meanwhile, unlike 2D dispersed stem cell therapy, the development of 3D culture platforms, including hydrogels, microfluidics, and biocompatible templates, to form 3D stem cell aggregates (e.g., spheroids) has shown transformative breakthroughs regarding tissue repair and regeneration.^[^ ^13^ ^]^ It is proven that 3D spheroids provide outstanding cell viability in harsh environments over in vivo wound areas and the hypoxic core of spheroids, escalating Hypoxia‐inducible factor 1‐alpha (HIF‐1α) protein expressions, which can activate vascular endothelial growth factors (VEGF), the most critical growth factor involved in angiogenesis.^[^ ^14^ ^]^ However, engineering challenges of the 3D spheroids therapy are in achieving: i) the labor‐intensive procedure for generating spheroids with uniform shape/size distribution; ii) facile delivery of sufficient spheroids with platforms that can extend over a large wound area; and iii) wireless spheroid modulation without any physical constraints on skin for clinical translation for widespread uses. Thus, a skin‐like, conformal patch device with functional therapeutic interfaces, capable of creating, delivering, and boosting 3D cells wirelessly over the wound area, is practically needed to address those shortcomings.

In this article, we present a wireless, reusable skin wound care platform designed for multimodal therapy, enabling synergistic photothermal–stem cell–light (PTSCL) treatment. The wireless PTSCL platform integrates four key features: i) polydimethylsiloxane (PDMS) patch with micro‐hole structures that support both the in situ formation and targeted delivery of 3D human adipose‐derived stem cell (hADSC) spheroids, which are advantageous for wound healing due to their abundance and ease of isolation^[^ ^15^ ^]^; ii) an Au‐coated nanoturf (Au/Nanoturf) membrane embedded within the PDMS patch, functioning as a photothermal interface for light‐to‐heat conversion,^[^ ^16^, ^17^ ^]^ thereby inducing mild photothermal‐hyperthermia at the wound site and spheroid for effectively upregulating wound healing effect of hADSCs; iii) a wireless light‐emitting diode (LED) array module powered via magnetic inductive coupling at a near‐field communication (NFC) frequency of 13.56 MHz, enabling on‐demand photothermal stimulation and light therapy with low‐level light irradiation (< 2.5 mW·cm^−2^); and iv) full reusability of the PTSCL patch platform, with preserved photothermal performance after repeated autoclave sterilization cycles, the safest and most reliable sterilization methods in clinical settings.^[^ ^18^ ^]^

In vitro experiments demonstrate that LED‐induced photothermal stimulation for 24 h preserves the high viability and biocompatibility of hADSC spheroids loaded within the patch. This continuous stimulation further enhances their therapeutic functionality, as evidenced by the significant upregulation of gene and protein markers related to angiogenesis and immunomodulation with a heat shock protein‐mediated mechanism. In contrast to electrical or laser‐based stimulation methods that require repeated or intermittent treatment until wound closure, the PTSCL platform enables effective therapy with a single 24 h‐light exposure. In vivo studies using freely moving animals demonstrated that this continuous stimulation, delivered via a wirelessly powered LED module integrated into custom‐designed cages, achieved therapeutic effects without anesthesia or behavioral restraint, while complying with human electromagnetic safety standards (4 W).^[^ ^19^ ^]^ PTSCL‐treated skin exhibited enhanced tissue maturation and revascularization across both the dermis and epidermis, outperforming other experimental groups, including only mild hyperthermia and LED therapy groups. Notably, complete wound closure was observed within approximately two weeks for large wound areas (2 cm‐by‐2 cm). Analysis of gene expression in the remodeled skin revealed substantial upregulation of key wound healing markers, including Fibronectin (3.8 folds), Keratin 14 (15 folds), Involucrin (4 folds), and cluster of differentiation 31 (CD31; 12 folds), compared to a control group treated with simple wound encapsulation (e.g., Tegaderm). These findings provide direct evidence of the synergistic effects of PTSCL therapy, highlighting it as an effective and simplified strategy for advanced skin wound care. In addition to these therapeutic outcomes, the Au/Nanoturf membrane‐embedded patch can be reused after standard autoclave sterilization without loss of performance. Such strategic engineering design and demonstrated biological efficacy, along with reusability, support its potential as a cost‐effective and practical wound management platform that could enhance the quality of care in clinical settings.

## 2 Results

### 2.1 Overall Design Strategies for Wirelessly‐Operated Synergistic PTSCL Therapy

**Figure 1:**
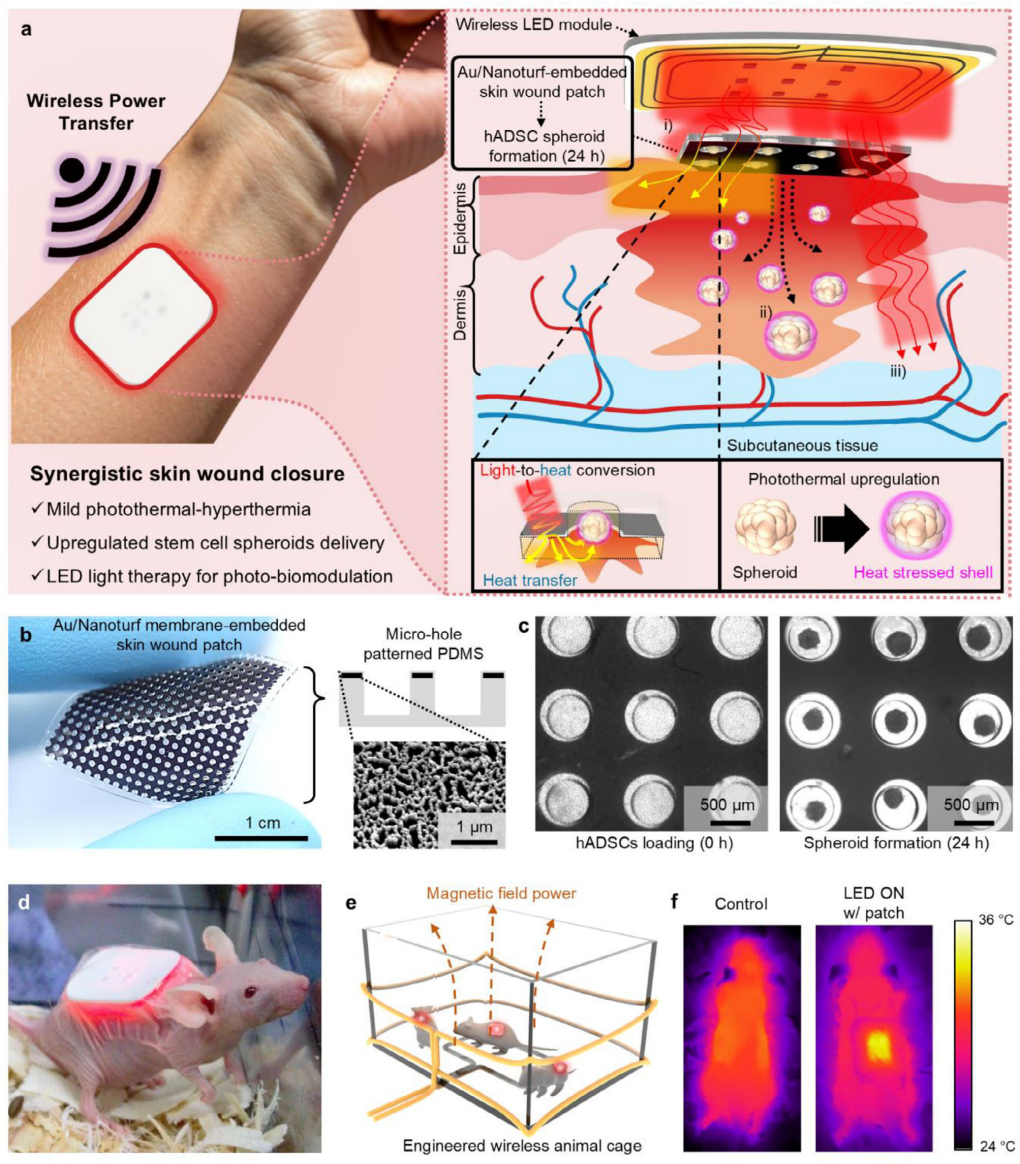
Overview of synergistic photothermal‐stem cell‐light (PTSCL) therapy for skin wound closure enabled by an Au/Nanoturf membrane‐embedded skin‐interfaced platform. a) Schematic illustration of the operating principle and functional layers of the wireless wound healing patch harnessing synergistic therapeutic effects. b) Photograph of an Au‐coated nanoturf membrane (Au/Nanoturf membrane)‐embedded PDMS patch with micro‐hole structures. A scale bar is 1 cm. Inset images show the cross‐sectional schematics of the patch and top‐view scanning electron microscopy (SEM) image of the Au/Nanoturf structures. c) Optical microscopic images of hADSCs loading (left; 0 h) and culturing (right; 24 h) to form the spheroids in the assembled patch. Scale bars are 500 µm. d) Representative photograph shows a skin‐mounted PTSCL platform integrated with a wireless light‐emitting diode (LED) module in a Balb/c nude mouse wound model. e) Schematic illustration of an engineered animal cage that can wirelessly operate LED modules by magnetic resonant coupling. f) Temperature distributions in an anesthetized mouse placed at the center of the animal cage with and without a skin‐interfaced wireless module.

**Figure** **1**a illustrates the comprehensive schemes of the wireless PTSCL patch platform. The PTCSL treatment begins with Au/Nanoturf membranes‐embedded PDMS patches loaded with hADSC spheroids (as depicted in the process flow diagram in Figure S1a, Supporting Information), which are applied to fully cover the wound area, followed by the placement of a wireless LED module. Upon wireless LED irradiation, the 630 nm light is converted into heat via the Au/Nanoturf membrane, generating mild photothermal‐hyperthermia. Simultaneously, this photothermal effect stimulates the spheroids within the micro‐hole patch, inducing a heat‐stressed shell that enhances their therapeutic potential. The activated spheroids are then delivered into the wound site, contributing to the remodeling and restoration of skin tissue integrity. By combining distinct therapeutic elements into a single platform, this wound healing approach aims to elicit interactive effects that collectively enhance tissue regeneration. As a result, the wireless PTSCL platform facilitates synergistic wound healing through a combination of i) mild photothermal‐hyperthermia, ii) delivery of photothermally upregulated hADSC spheroids, and iii) wireless LED light therapy (Figure S1b, Supporting Information).

The process for this synergistic approach began with preparing an Au/Nanoturf‐embedded PDMS patch. In previous studies,^[^ ^16^, ^17^, ^20^ ^]^ a randomly disordered, high aspect ratio (AR) sub‐micrometer structure, known as nanoturf, displayed distinctive optical properties after Au deposition on a nanometer scale, enabling light‐to‐heat conversion in a thin (40 µm), free‐standing membrane. Due to its readily structural transformation of a photocurable polymer (Polysiloxane acrylate; PSA) through unconventional lithography (Details provided in the Experimental Section and Figure S2, Supporting Information), the scalable membrane^[^ ^16^ ^]^ can be seamlessly extended to cover the wound area. The inset SEM image in Figure 1b (right bottom) presents the morphological integrity and stability of Au/Nanoturf structure. The micro through‐holes (600 µm in diameter) of free‐standing Au/Nanoturf membrane were positioned through the SU 8 negative photoresist micro‐pillars (500 and 300 µm in diameter and height, respectively) onto the Si wafer (Figure S3, Supporting Information). Subsequent thermal curing and demolding of the PDMS precursor created the Au/Nanoturf‐embedded PDMS patch (2 cm‐by‐2 cm) micro‐holes for hADSC loading (Figure 1b; The diameter and height of a micro‐hole and PDMS thickness are 500, 300, and 500 µm, respectively). Figure 1c presents sequential bright‐field images illustrating the loading and culturing of hADSCs within the assembled patch, leading to 3D spheroid formation in 24 h. The dark region in the image corresponds to the Au/Nanoturf membrane, which absorbs incident light, whereas the brighter regions indicate the cell‐loaded micro‐holes. Furthermore, such optical transparency of PDMS allows for additional LED light stimulation of the loaded cells, while also permitting light penetration to the underlying wound tissue to induce light therapy, commonly called photo‐biomodulation (PBM).

To verify the therapeutic efficacy of the PTSCL therapy, in vitro and in vivo experiments were performed via various assays and analysis of cells and remodeled skin tissues. Figure 1d shows a representative photograph of the wireless PTSCL platform applied to a nude mouse wound model with a wound area of 2 cm‐by‐2 cm. Animal models are freely moved, throughout the PTSCL therapy, inside an engineered animal cage that generates uniform non‐radiative magnetic field at an operating frequency of 13.56 MHz (Figure 1e). Figure 1f compares temperature distributions in an anesthetized mouse placed at the center of the animal cage with and without a skin‐interfaced platform.

### 2.2 Characterization of the Au/Nanoturf Membrane‐Embedded PDMS Patch

**Figure 2:**
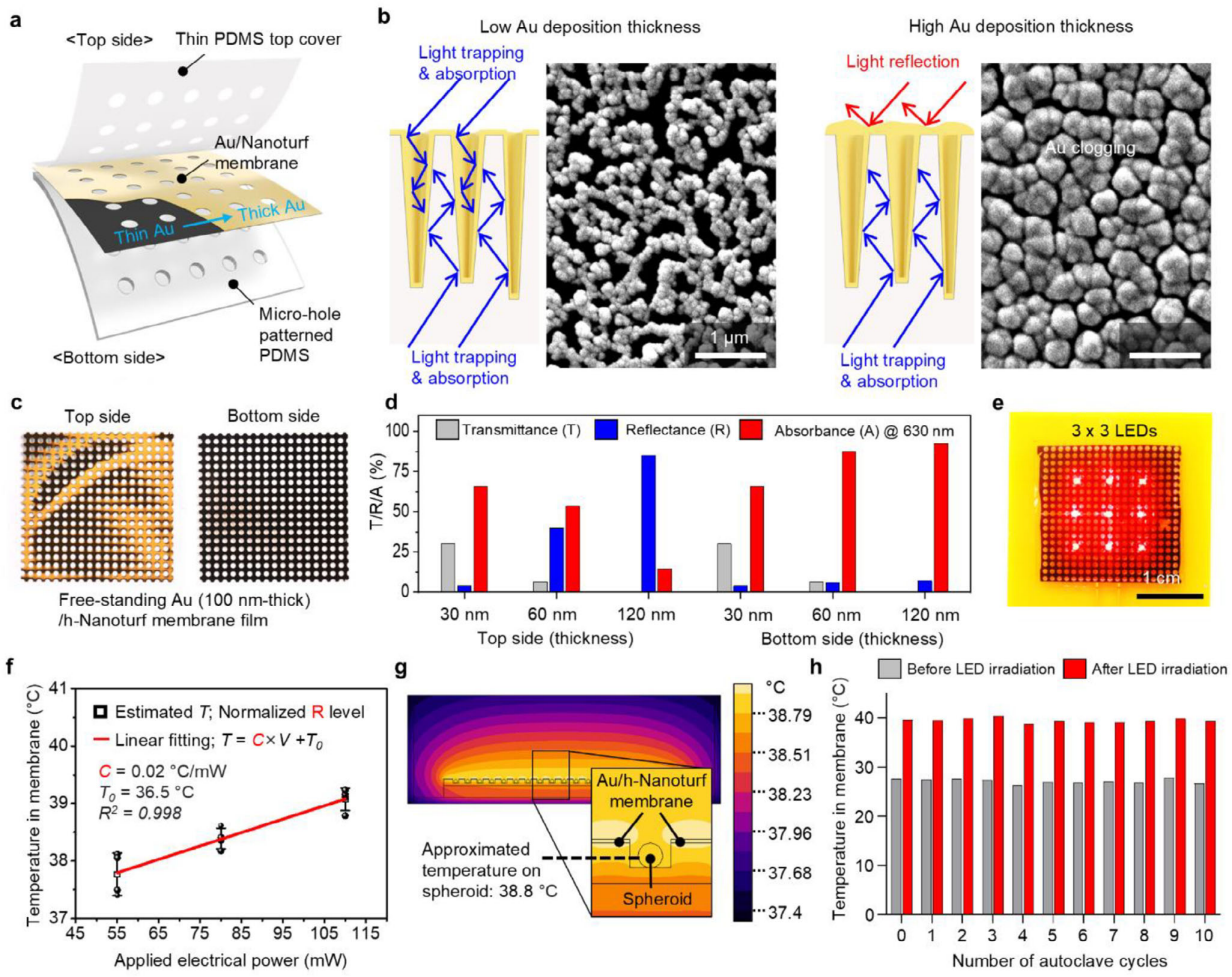
Characterizations of an Au/Nanoturf membrane‐embedded skin wound patch. a) Exploded‐view illustration of the Au/Nanoturf membrane‐embedded PDMS patch with micro‐hole structures. b) Cross‐sectional schematics and SEM images of Au/Nanoturf structures with relatively thinner (left; 30 nm‐thick) and thicker (right; 120 nm‐thick) Au deposition. c) Top (left) and bottom (right) side images of a free‐standing 120 nm‐thick Au/Nanoturf film (2 cm‐by‐2 cm) are shown. d) Optical properties with respect to the top and bottom sides of Au‐coated nanoturf film according to the Au deposition thickness ranging from 30 to 120 nm at 630 nm‐wavelength light. e) Photography of the Au/Nanoturf membrane‐embedded skin wound patch placed on the 3 × 3 LED array module (630 nm‐wavelength). A scale bar is 1 cm. f) Calculated temperature profile in the membrane of the skin wound patch immersed in a phosphate‐buffered saline (PBS) solution at 36.5 °C (*T_0_*) as a function of applied electrical power in the LED module. g) Finite element analysis (FEA) result of the temperature distribution of the spherical cells‐loaded patch immersed into the cell culture media (36.5 °C) at a certain temperature (39 °C) of the Au/Nanoturf membrane. h) Effect of repeated autoclave sterilization on photothermal heating performance of an Au/Nanoturf‐embedded patch.

**Figure** **2**a shows the exploded‐view illustration of the Au/Nanoturf membrane‐embedded patch through processing steps described in Figure S3 (Supporting Information). To efficiently induce mild photothermal‐hyperthermia and deliver LED light to the wound area through the Au/Nanoturf membrane, the bilateral optical properties of the membrane were tuned by adjusting metal deposition thickness (Figure S4, Supporting Information). With the thin Au deposition (less than 60 nm) onto the nanoturf membrane via sputtering system, the randomly distributed geometries at the sub‐wavelength scale possess unique black coloration due to the tapered cross‐sectional structure for light trapping (Figure 2b; left schematics, blue arrows: light can be trapped or absorbed into the structures), inducing localized surface plasmon resonance (LSPR) around the metallic nanostructures with a broad range of resonance wavelength. In contrast, as Au deposition thickness increases (more than 100 nm), the top surface openings of the nanoturf structure are gradually clogged (Figure S5, Supporting Information), thereby enhancing the inherent reflective optical properties of the deposited metal (i.e., gold color) as shown in Figure 2b (right schematics, red arrows: reflected light from the surface), and Figure S4 (Supporting Information). Interestingly, the bottom side of the nanoturf membrane exhibits a deeper black color with increasing Au thickness owing to enhanced light absorption as a consequence of minimized loss from optical reflection and transmission. Figure 2c displays photographs of the top and bottom sides of the free‐standing Au/Nanoturf membrane with a deposition thickness of 120 nm, showing a notable difference in the bilateral optical properties. Figure 2d presents the optical properties, including transmittance, reflectance, and absorbance (T/R/A) of the membrane, as a function of metal deposition thickness on both sides. Reflectance on the top side of the membrane increased to 85% with a 120 nm‐thick Au layer, while absorbance on the bottom side increased and saturated ≈94%. These distinct bilateral optical characteristics were strategically exploited in the PTSCL therapy: i) the bottom surface facilitated efficient light‐to‐heat conversion upon LED irradiation, inducing mild photothermal‐hyperthermia; and ii) the top surface enhanced multiple light reflections, thereby maximizing light delivery due to reflection and scattering at the skin interface.

To validate the photothermal hyperthermia resulting from light‐to‐heat conversion by the Au/Nanoturf membrane, the assembled PDMS patch was placed onto a 3 × 3 630 nm‐LED array module (Figure 2e; Figure S6, Supporting Information). Temperature rise within the micro‐holes for photothermal upregulation of spheroids was assessed by filling the holes with temperature‐sensitive thermochromic pigments, allowing visualization of temperature distribution under varying electrical power applied to the LED module. These applied powers corresponded to the light intensities between 1.01 and 2.40 mW·cm^−2^ (Figure S7, Supporting Information). The patch was immersed in a phosphate‐buffered saline (PBS) solution at 36.5 °C (*T_0_*) in a culturing plate. Figure S8a,b (Supporting Information) shows the variation of extracted RGB level with increasing temperature ranging from 36 to 42 °C, serving as a reference for temperature visualization. Figure 2f and Figure S8c,d (Supporting Information) present an estimated temperature elevation (between 37 and 39 °C) as the applied electrical power of LED modules ranging from 54 to 119 mW. Finite element analysis (FEA) of the temperature distribution in the spheroid‐loaded micro‐hole patch was performed under a simulation condition in which the Au/Nanoturf membrane was set to 39 °C. FEA results showed the temperature around the spheroid within the micro‐holes reached ≈38.8 °C (Figure 2g; Figure S9, Supporting Information), indicating that photothermal heating from the membrane can effectively influence the hADSC spheroids, promoting their upregulation. Notably, the Au/Nanoturf membrane‐embedded patch can withstand repeated autoclave sterilization cycles (10 times; Figure 2h) without compromising its photothermal performance. This reusable performance ensures reliable therapeutic efficacy while enhancing clinical practicality and cost‐efficiency.

### 2.3 Temporal Effects of Mild Photothermal Hyperthermia on Spheroids Within the Patch

**Figure 3:**
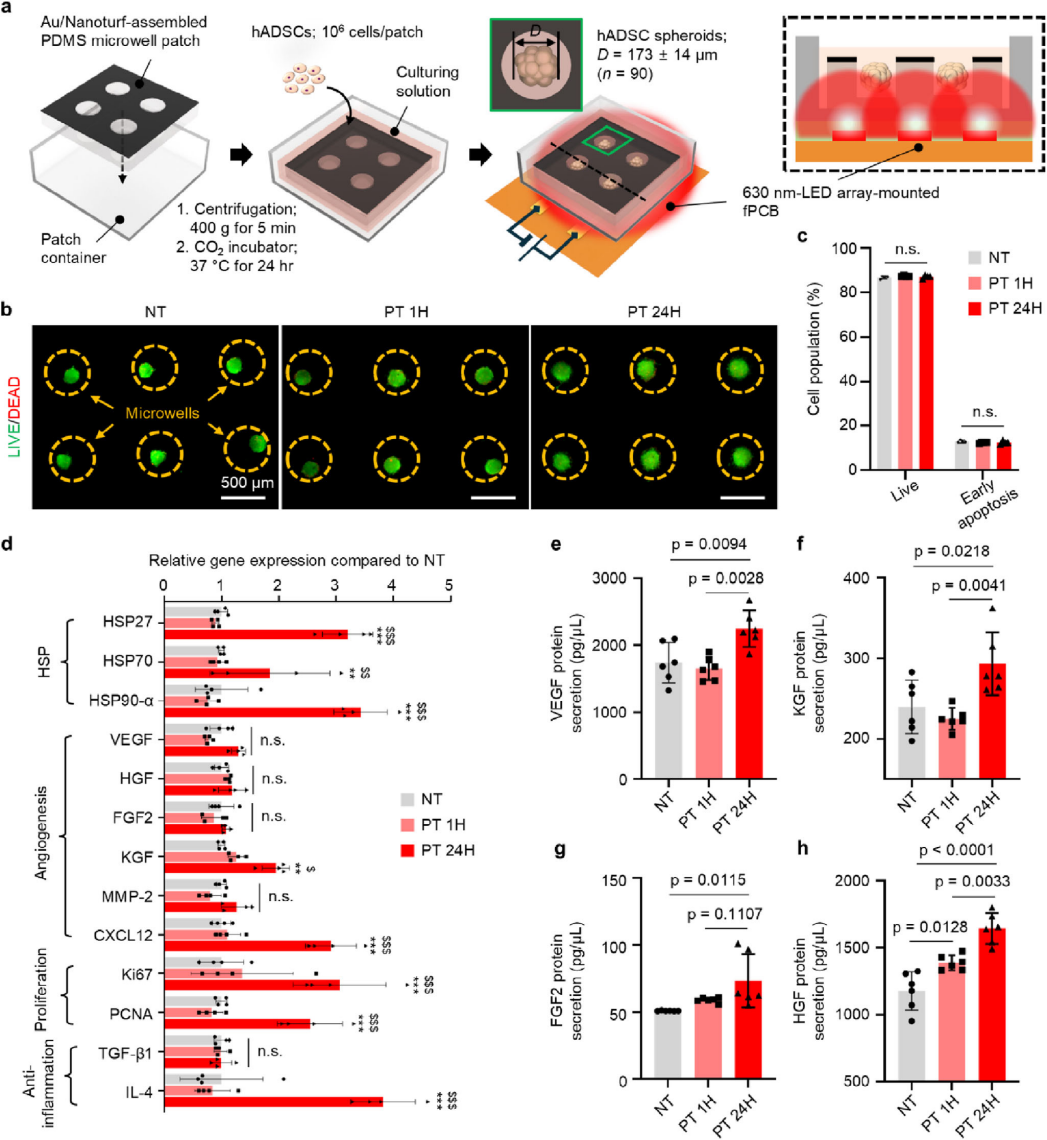
In vitro cell viability and gene/protein evaluation of photothermally upregulated hADSCs spheroids within the patch. a) Schematic illustrations of the formation and photothermal upregulation of hADSCs spheroids (D = 173 ± 14 µm; mean values ± s.d.; sample size = 90) within the patch under irradiation of the LED array (3 × 3, 630 nm). Inset schematics show the cross‐sectional view of hADSCs spheroids‐loaded patch along with the LED array inside the container filled with the culturing solution. b) Representative results of live (fluorescein diacetate (FDA); green) and dead (ethidium bromide (EB); red) assay of no treatment (NT), photothermal stimulation for 1 h (PT 1H), and 24 h (PT 24H) groups. Scale bar is 500 µm. c) Cell viability evaluated with flow cytometry double staining of Annexin V and 7‐Aminoactinomycin D (7‐AAD) (Sample size = 4). d) Comparison of gene expressions in NT, PT 1H, and PT 24H group evaluated by quantitative reverse transcription PCR (qRT‐PCR) (Sample size = 4, one‐way ANOVA, ^**^P< 0.01, ^***^P< 0.001 compared to NT, ^$^P < 0.05, ^$$^P < 0.01, and ^$$$^P < 0.001 compared to each group). Evaluated genes are associated with factors of heat shock protein (HSP), angiogenesis, proliferation, and anti‐inflammation. e–h) Amount of vascular endothelial growth factor (VEGF; e), keratinocyte growth factor (KGF; f), fibroblast growth factor (FGF2; g), hepatocyte growth factor (HGF; h) secreted from hADSCs spheroids in each group as evaluated by enzyme‐linked immunosorbent assay (ELISA, Sample size = 6, one‐way ANOVA).

To evaluate the temporal effects of photothermal hyperthermia for hADSC spheroids, induced by the Au/Nanoturf membrane, the duration of photothermal stimulation was manipulated at 0 h (NT), 1 h (PT 1H), and 24 h (PT 24H), corresponding to short‐ and long‐term stimulation groups, respectively (see Figure S10, Supporting Information for detail). **Figure** **3**a shows the experimental setup to estimate cell viability, gene, and protein expression of spheroids for each group. An assembled patch was placed into each square wall container (width, length, and height of 2.1, 2.1, and 1 cm, respectively) for stem cell culturing. The loaded number of hADSCs per patch was ≈10^6^ cells, which were subsequently centrifuged and incubated in a CO_2_ incubator for 24 h to form the spheroids (for details, See Experimental Section). As shown in Figure 3a and Figure S11a (Supporting Information), the irradiation of LED array, powered from DC supply for inducing photothermal effect in the patch began right after spheroid formation (average diameter (D) of the spheroids = 173 ± 14 µm, n = 90; Figure S12, Supporting Information), yielding mild hyperthermia to the spheroids at specific temperature ranging from 38 to 39 °C.

Biocompatibility of the assembled patch and spheroid viability following photothermal stimulation were assessed using live/dead staining (green: live cells and red: dead cells; Figure 3b), terminal deoxynucleotidyl transferase dUTP nick end labeling (TUNEL) assay (blue: cell nuclei and green: dead cells; Figure S11b, Supporting Information), and flow cytometry (Figure 3c). Morphological results regarding live/dead staining and the fluorescence signal density of PT 1H and 24H groups were similar to those of the NT group. Additionally, all experimental groups showed rare green signals (dead cells) in the core section of each spheroid in the TUNEL assay (Figure S11b, Supporting Information). In flow cytometry, as a quantitative analysis, stained with Annexin V and 7‐aminoactinomycin D (7‐AAD) for profiling cell viability, inducing mild hyperthermia ranging from 0 to 24 h does not affect cell viability of stem cell spheroids (Figure 3c; Figure S13, Supporting Information). Multiple viability assays consistently showed no significant differences, suggesting that mild photothermal‐hyperthermia does not compromise the viability of stem cell spheroids.

Next, quantitative real‐time polymerase chain reaction (qRT‐PCR) analysis reveals relative changes in mRNA expression levels of genes associated with heat shock response, angiogenesis, and cell proliferation (Figure 3d). Among the analyzed genes, HSP27 and HSP90‐α showed more than a 3 fold increase, while HSP70 increased by ≈1.8 fold in the PT 24H group compared to other groups. Specifically, HSPs are essential to heal wound areas because they play significant roles, including inflammation, biological debridement, cell proliferation/migration, and collagen synthesis.^[^ ^21^ ^]^ Accordingly, precisely controlled photothermal stimulation, yielding mild hyperthermia to stem cells by controlling LED light intensity, wavelength, and energy density, makes effective upregulation of growth factor secretion signaling without damaging stem cells.^[^ ^22^ ^]^ Gene expression levels related to angiogenesis, including VEGF, KGF, MMP‐2, and CXCL12, were upregulated after 24 h of photothermal stimulation. Consistently, enzyme‐linked immunosorbent assay (ELISA) analysis confirmed increased secretion of angiogenic paracrine factors, with protein levels of VEGF, KGF, FGF2, and HGF elevated by 1.29, 1.22, 1.44, and 1.68 folds, respectively, in the PT 24H group compared to the NT group (Figure 3e–h). Such enhanced secretion of these paracrine factors can contribute to accelerated angiogenesis and tissue remodeling at the wound area.^[^ ^23^, ^24^ ^]^

Meanwhile, cell proliferation markers (Ki67 and PCNA) and anti‐inflammatory cytokines (TGF‐β1 and IL‐4) were also upregulated in the PT 24H group. Hierarchical clustering heatmap analysis showing a clear separation between the PT 24H group and the other groups (Figure S14, Supporting Information) further supports that sustained photothermal stimulation for 24 h markedly changes the expression of genes in hADSC spheroids. Although previous studies have reported that hyperthermia above 39 °C can reduce cell proliferation due to thermal damage,^[^ ^25^, ^26^ ^]^ the collected results demonstrate that mild photothermal‐hyperthermia enhances the proliferative capacity of hADSCs. This effect may be attributed to the gradual upregulation of heat shock proteins (HSPs) under non‐cytotoxic, mild hyperthermic conditions for 24 h, which helps maintain cellular homeostasis and promotes cell proliferation.^[^ ^27^ ^]^ Ultimately, the promoted proliferation may improve engraftment efficiency by providing a more robust and resilient cell population, thereby enhancing therapeutic efficacy.^[^ ^28^ ^]^

### 2.4 Synergistic Activation of Therapeutic Pathways in Photothermally Stimulated Spheroids

**Figure 4:**
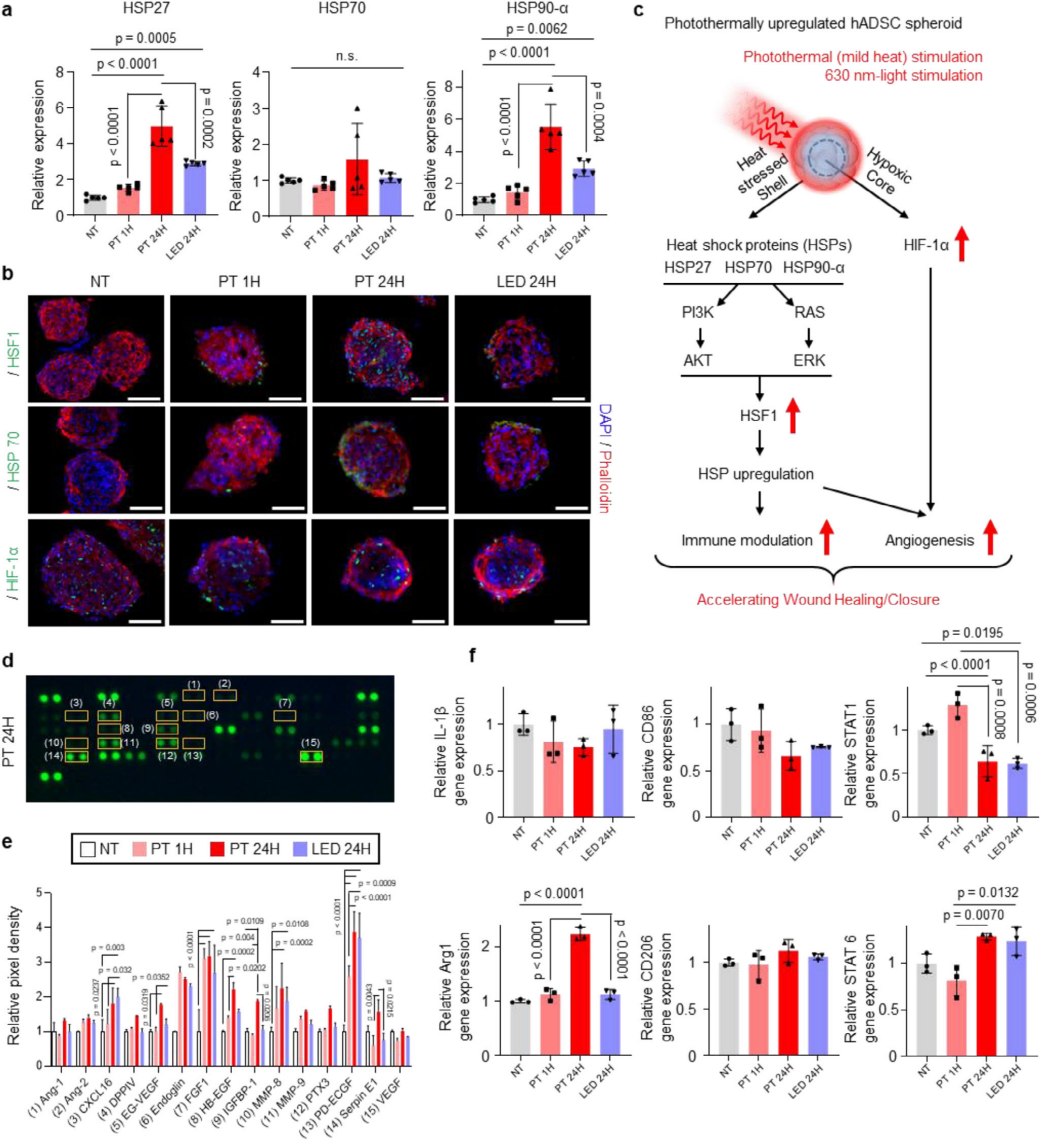
In vitro tests of photothermally upregulated hADSC spheroids to verify enhanced factors associated with immune modulation and angiogenesis. a) Comparison of the heat shock pathway related HSP 27, HSP 70, and HSP 90‐α gene expression in each group (no treatment (NT), photothermally upregulation for 1 h (PT 1H), and 24 h (PT 24H) and only LED light‐induced upregulation (LED 24H) via qRT‐PCR (Sample size = 5, one‐way ANOVA). b) Representative immunofluorescence images of the heat shock related protein expression in each group (DAPI; blue., phalloidin; red and HSF1 (top), HSP70 (middle), HIF‐1α (bottom); green). Scale bar is 100 µm. c) Pathway illustration of the intracellular signaling pathway of the hADSCs spheroid upregulated by photothermal and 630 nm‐light stimulation, yielding enhanced immune modulation and angiogenesis. d) Representative angiogenesis array dot blot image of the PT 24 group. e) Relative pixel density of angiogenesis array in each group, compared to the NT group (Sample size = 2, two‐way ANOVA). f) Evaluation of M1‐to‐M2 polarization effect of condition media (CM) derived from each group by treatment of M1 macrophage (Sample size = 3, one‐way ANOVA).

Due to the optical transparency of the micro‐hole matrix in the Au/Nanoturf membrane‐embedded PDMS patch, 630 nm‐LED light can directly reach the spheroids during irradiation, allowing both photothermal and direct light stimulation to occur simultaneously. To isolate the therapeutic effect of light stimulation alone from that of photothermal effects under identical LED conditions, an additional group (LED 24H, Figure S10, Supporting Information) using a PDMS patch without the Au/Nanoturf membrane was included in the following study. As shown in **Figure** **4**a, while gene expression levels of HSP27, HSP70, and HSP90‐α were slightly elevated in the LED 24H group, they were significantly lower than those in the PT 24H group. For spatial analysis of the heat shock‐related protein expression, Figure 4b presents representative immunofluorescence images of key proteins such as heat‐shock factor 1 (HSF1; top), HSP 70 (middle), and HIF‐1α (bottom). HSF1 and HSP 70, which are associated with the upregulation of paracrine factor secretion, in the PT 24H group, exhibit the most positive signals among all examined groups. In this group, HSF1 is uniformly distributed across the spheroid (top; Figure 4b). On the contrary, there is no significant difference between the PT 1H and the LED 24H groups. In the PT 24H group, HSP70 was mainly expressed in the outer shell of the spheroid, corresponding to the region directly affected by heat stress. The LED 24H group showed a slight increase in the same region, but the signal was weaker than in the PT 24H group (middle; Figure 4b). Meanwhile, hypoxia‐inducible factor‐1α (HIF‐1α), involved in promoting angiogenesis, was consistently observed within the spheroids across all experimental groups due to the hypoxic condition after spheroid formation (bottom; Figure 4b). Collectively, these results suggest that the integrated structural design, facilitating both photothermal and LED light stimulation, plays a crucial role in synergistically enhancing the biological activity of 3D hADSC spheroids, particularly through mechanisms involving Hif‐1α and HSP‐mediated signaling.

Western blot analysis verified the activation of the AKT and ERK signaling pathways exclusively in the PT 24H group, as evidenced by the presence of phosphorylated AKT and ERK1/2 (p‐AKT and p‐ERK1/2; Figure S15, Supporting Information). Although HSF1 and HSP70 remained activated for up to 24 h after photothermal stimulation (Figure S16, Supporting Information), their expression levels were markedly reduced following treatment with AKT or ERK inhibitors (Figure S17, Supporting Information). Furthermore, pre‐treatment with inhibitors targeting ERK, AKT, and HIF‐1α prior to PT 24H stimulation significantly suppressed the secretion of angiogenesis‐related paracrine factors (Figure S18, Supporting Information). Both AKT and ERK inhibition resulted in a significant reduction in HSP70 and HSF1 expression compared to the non‐inhibited PT 24H group, suggesting that these pathways are indeed essential for HSP upregulation in response to photothermal stimulation. Also, our results demonstrate that HIF‐1α plays a central and indispensable role in mediating angiogenesis in photothermally stimulated ADSC spheroids. Based on in vitro findings, we propose that the upregulated HSPs in stimulated spheroids contribute to two key therapeutic mechanisms: i) enhanced wound healing via activation of protein kinase B (PKB, or AKT) and extracellular‐signal‐regulated kinase (ERK) signaling pathways; and ii) increased release of angiogenic paracrine factors, in coordination with HIF‐1α, typically expressed in the hypoxic core of spheroids that is essential for angiogenesis (Figure 4c). These results suggest that HSP upregulation is mediated through the AKT and ERK pathways, thereby contributing to the enhanced secretion of angiogenic paracrine factors along with Hif‐1α activation. Specifically, synergistic activation of HSF1 contributes to both the angiogenic potential of stem cell spheroids and the upregulation of heat shock proteins,^[^ ^29^, ^30^ ^]^ driving their expression predominantly in the outermost shell of hADSC spheroids. This elevated HSP expression promotes the secretion of angiogenesis and immunomodulatory‐related proteins,^[^ ^27^, ^31^, ^32^ ^]^ enhancing therapeutic effects by accelerating wound healing and closure.

An additional analysis of angiogenesis‐related protein secretion was conducted using a human angiogenesis array (Figure 4d,e; Figure S19, Supporting Information). Among specific target proteins in the kit, we selectively identified 15 proteins (labeled from (1) to (15)), exhibiting differential expression levels with a bar graph by calculation of the pixel density of each protein normalized by each of the reference spots and compared to the NT group. Overall, the PT 24H group demonstrated the most diverse pro‐angiogenesis protein secretion, while some proteins exhibited higher expression in each group. Moreover, the human umbilical vein endothelial cells (HUVECs) tube formation assay, which evaluates the later stage of angiogenesis, confirmed by 3D capillary‐like tubular structures, was introduced. PT 24H group‐derived conditioned medium (CM) treated HUVEC formed the most tubes among the groups. Results in Figure 4d,e, and Figure S20 (Supporting Information) indicate that upregulated spheroids in the PT 24H group had the highest potential for the angiogenic process. In the fibroblast migration assay, the group treated with CM from the PT 24H group exhibited the most effective in vitro wound closure (Figure S21, Supporting Information). This suggests that PT 24H treatment significantly promotes fibroblast migration, indicating its strong potential in enhancing wound closure.

The immunomodulatory effects of photothermally upregulated spheroids needed to be confirmed by assessing the M1(pro‐inflammatory macrophages)‐M2 (anti‐inflammatory macrophages) polarization shift, which represents the transition of the wound healing process from the inflammatory to the tissue repairing phase.^[^ ^33^ ^]^ To test pro‐ to anti‐inflammation transition effect of each group, CM was retrieved from each group and treated with M1 macrophage. Here, representative M1 and M2 macrophage markers (M1; IL‐1β, CD86, and Signal transducer and activator of transcription 1 (STAT1) and M2; Arginase‐1 (Arg1), CD206, and STAT6, respectively) were detected by qRT‐PCR (Figure 4f). At the phase of M1 macrophages, the PT 24H group exhibited relatively lower expression level of each gene than those of other groups (top; Figure 4f). The LED 24H group showed a higher expression than the PT 24H group, despite showing downregulation of the M1 marker compared to the NT and PT 1H groups. In contrast, at the M2 macrophage stage, the PT 24H group showed the highest expression for each gene (bottom; Figure 4f). Notably, the expression level of Arg1 was significantly increased in the PT 24 group. It means that an effective M1‐M2 polarization shift of the spheroid occurs during mild hyperthermia induced by photothermal stimulation.

### 2.5 Design Strategies for the Wireless Module and Uniform Power Distribution

**Figure 5:**
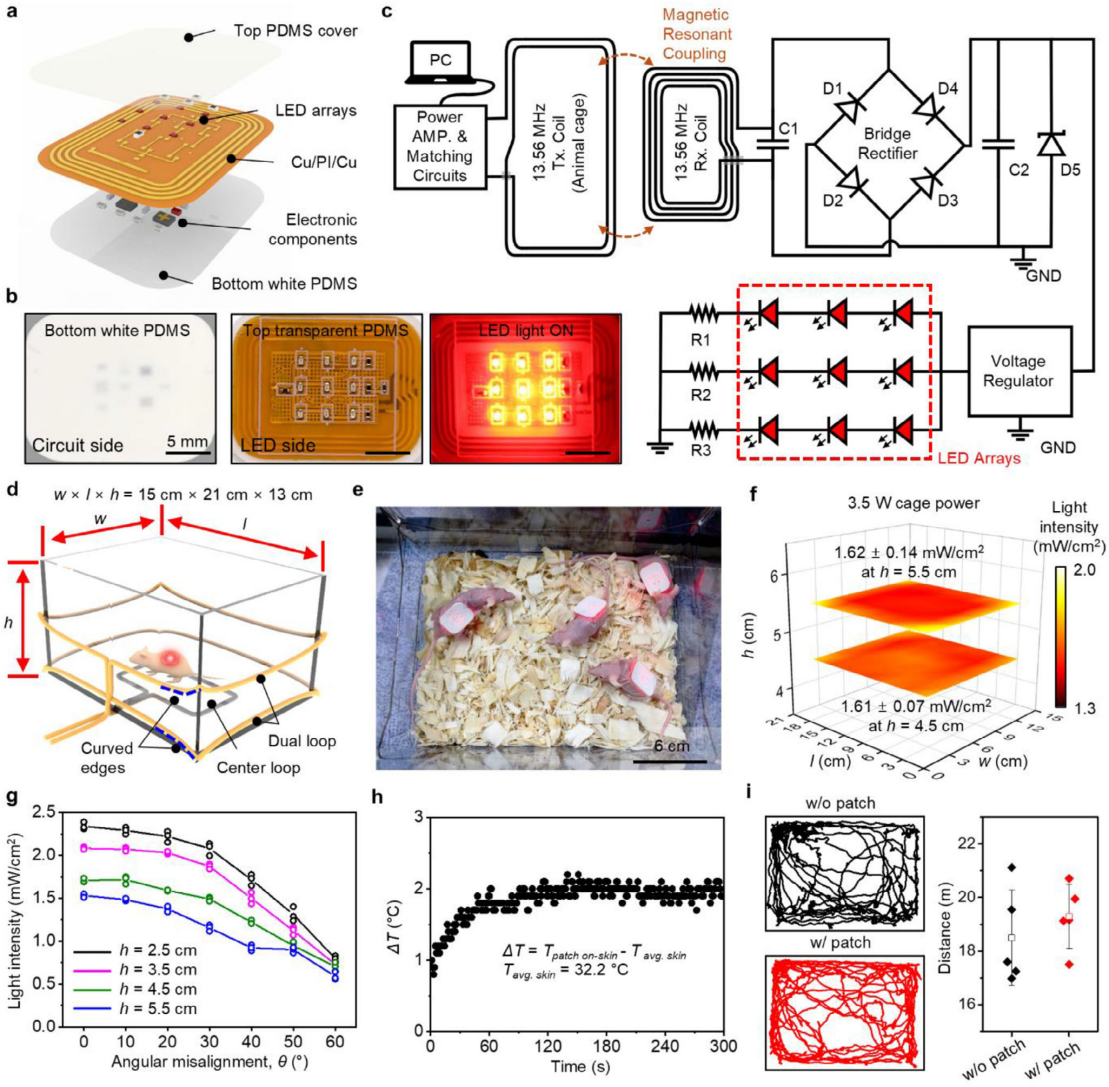
A skin‐interfaced wireless LED module and engineered animal cage for efficient, uniform wireless power transmission. a) Exploded‐view illustration of the constituent layers of the wireless module: top transparent PDMS cover layer, functional electronics, and bottom PDMS cover layer mixed with white silicone dye. b) Photographs showing the bottom (left) and top (center) sides of the module while the LED array was turned on/off (right). c) Schematic circuit and block diagrams of the wireless module to harvest near‐field communication (NFC) frequency (13.56 MHz) power transmitted from an engineered animal cage. d) Schematic illustration of the engineered animal cage, including a center loop at the bottom and dual loops through the sidewalls with curved edges for uniform field distribution. The center loop enhances the magnetic field at the center of the cage, whereas the curved edges in each corner decrease the localized magnetic field stemming from the edge effect. e) Photograph of freely moving mice wearing the lightweight skin‐interfaced wireless module powering LED arrays mounted on a skin wound. f) The distribution of the light intensity at 630 nm wavelength in each compartmentalized area (3 cm × 3 cm; mean values ± s.d.; sample size = 3) with different heights (h) at a radio frequency (RF) input power of 3.5 W. g) Variations of the light intensity as a function of angular misalignment (*θ*) for different *h* (mean values ± s.d.; sample size = 3). h) Temperature difference (*ΔT*) between the patch on the skin and the skin temperatures of an anesthetized mouse at the center of the cage with RF powering. i) Representative trajectory plots (left) and calculated total travel distance (right) of the mice mounted with (red) and without (black) the wireless module in the cage (sample size = 5).

The proposed wireless LED module comprises a coil antenna and tuning capacitors to match the resonance frequency for optimal wireless coupling with the transmitting signal at a near‐field regime, the rectification unit, the power regulation unit, and the load in the form of an LED array. To maintain a viable device footprint in reference to the target subject model, monolithic multilayers are designed utilizing a bilayer coil antenna (double‐layered coils with 4 turns, *R_DC_* = 1.7 Ω, *L* = 7.27 µH, *Z_real_* = 60.72  Ω, self‐resonance frequency = 15.96 MHz, and Q‐factor = 10.2, for details, see Figure S22, Supporting Information) accompanied by surface mount functional units at the bottom and the load at the top side of the flexible printed circuit board (fPCB) as illustrated in **Figure** **5**a. Furthermore, we devised two types of soft PDMS encapsulates (Figure 5b); a combination of top transparent cover and bottom white layer, to efficiently deliver LED light into the wound site, while minimizing light loss through the back circuit side (Figure S23a, Supporting Information). The white encapsulation on the back circuit side also enhances light intensity on the top LED side through the reflection (Figure S23b, Supporting Information). The overall circuit and block diagram of the wireless system is shown in Figure 5c where 13.56 MHz wireless power transmitted through the cage antenna is harvested by the wireless LED module by a magnetic resonant coupling mechanism. This power consideration is critically important in the proposed module comprising a 3×3 LED array as a load. Besides, to ensure optimal power transfer efficiency in near‐field operations, where loading conditions can significantly impact the performance, the transmitting circuit is tuned by adjusting its tuning capacitor and resistor according to the number of loads (Figure S24, Supporting Information). At the receiver module, we utilized a bridge rectifier configuration so that the harvested AC power can be efficiently rectified by using both positive and negative halves of the signal, and possesses simple circuitry and minimized power loss compared to the standard full‐wave rectifier that requires a center‐tapped transformer. Furthermore, the Zener diode (D5 in Figure 5c) acts as an overvoltage protection unit that effectively minimizes circuit heating, specifically induced when the regulator is overloaded. Thus, through the wireless LED module, we were able to harvest sufficient operating power (≈75 mW) at the load by supplying 3.5 W power to the transmitting cage antenna uniformly. Indeed, the power supply complies with the power regulation (4 W) suggested by the Federal Communications Commission (FCC) for human exposure.^[^ ^19^ ^]^

The conventional cage antenna designed for transmitting wireless power incorporates regular intertwined loop coils^[^ ^34^, ^35^ ^]^ but fails to maintain a uniform power distribution within the cage (for details, see Figure S25, Supporting Information). Indeed, the power is concentrated at the corners of the cage because of the edge effect, leading to higher field intensity and decreasing toward the center (Figure S22c, Supporting Information). To overcome this effect, we engineered a transmitting coil design (Figure 5d,e; Figure S26, Supporting Information) in such a way that the concentrated field intensity on the edges is minimized by an augmented conductive pathway directed off the field along the z‐axis, whereas the power deficiency at the center is circumvented by the additional bottom loop at the center covering nearly one forth (24.76%) of the cage area (width and length of 15 and 21 cm, respectively). While power stability can also be achieved by using a relatively bulky supercapacitor and boost regulators at the wireless module, we maintained uniform light irradiation by optimizing the cage antenna. This strategy enables reducing the number of electronic components, resulting in a thinner and lighter module to apply for small animal subjects. Thus, optimizing the cage antenna yields a nearly uniform light intensity of ≈1.61 ± 0.07 mW·cm^−2^ and 1.62 ± 0.14 mW·cm^−2^ at vertical heights (*h*) of 4.5 and 5.5 cm, respectively (Figure 5f; Figure S27, Supporting Information). The physical misalignment of the wireless LED module, typically about the generated magnetic field, is imperative in the freely moving mouse and is therefore responsible for minimizing the power harvesting efficiency. In this regard, our study suggests that there is not a significant drop in the light intensity up to an angular misalignment of 30° at a vertical height ranging from 2.5 to 5.5 cm (Figure 5g; Figure S28, Supporting Information). We further examined the photothermal effect on the anesthetized mouse with radio frequency (RF) powering (3.5 W), both with (i.e., patch on‐skin temperature) and without the wireless patch module (i.e., skin temperature), and found an elevation in temperature (*ΔT*) of ≈2 °C from an average skin temperature of 32.2 °C, approaching saturation within 90 s of exposure (Figure 5h; Figure S29, Supporting Information), which is able to induce mild hyperthermia. Moreover, as is evidenced by the trajectory study (Figure 5i), the developed patch did not exhibit any significant limitations in the natural kinematics of the freely moving mouse (18.5 ± 1.78 m without patch (black) and 19.29 ± 1.19 m with patch (red), respectively (n = 5)), suggesting a viable model for the proposed application.

### 2.6 Improved Skin Wound Healing Effect of PTSCL Therapy

**Figure 6:**
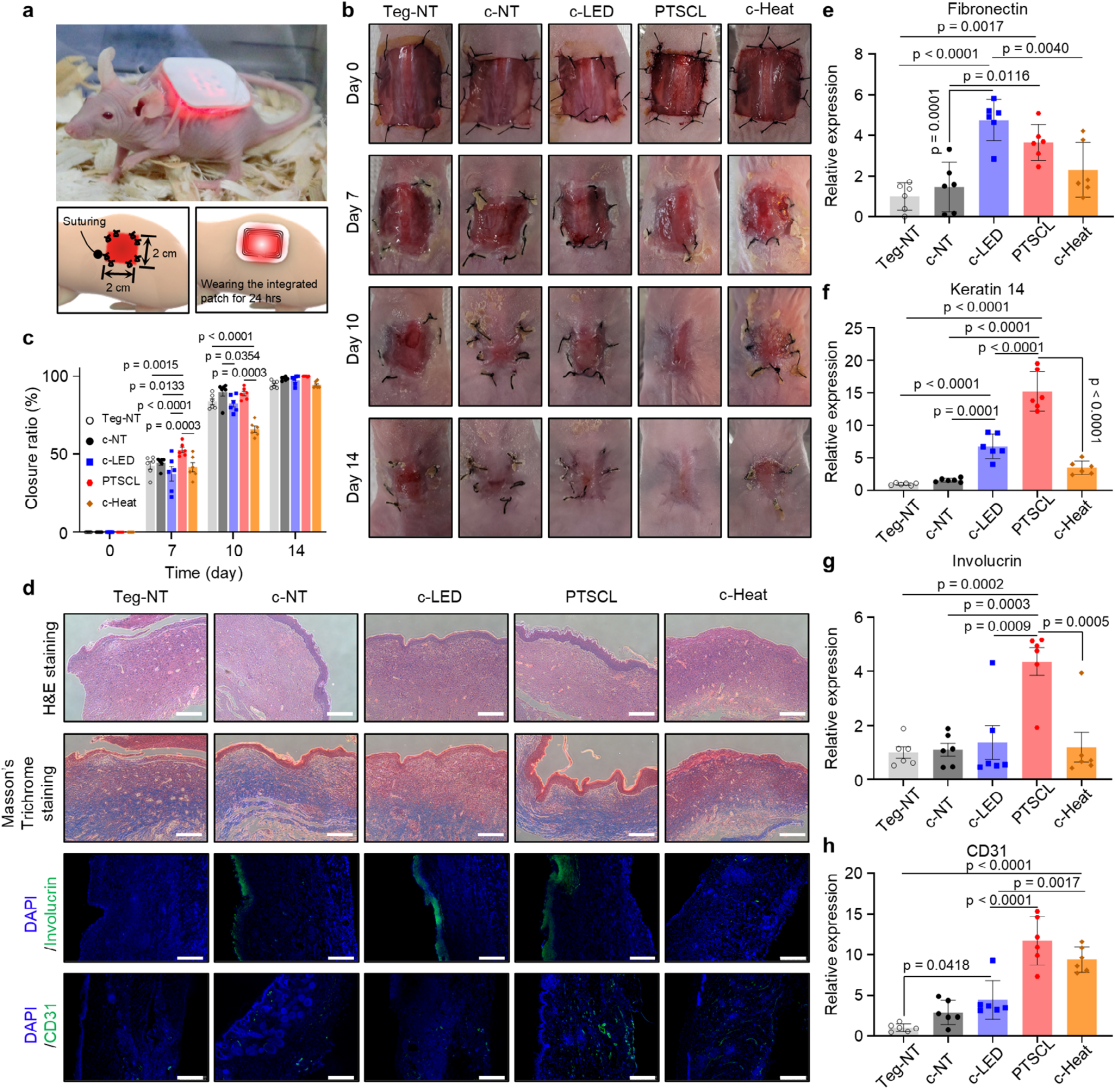
In vivo wound healing efficacy of the wireless PTSCL platform for synergistic skin wound closure. a) Photograph and schematic illustrations of the mouse model wearing the integrated skin wound healing patch for 24 h. Surgical sutures (each side of the 2 cm x 2 cm wound dimension) are used to prevent skin contracture. b) Representative photographs of sequential skin wound closure for 14 days in each group (no treatment without hADSCs; Teg‐NT, no treatment with hADSCs; c‐NT, LED treatment with hADSCs; c‐LED, photothermal treatment with hADSCs; PTSCL, and thermal treatment with hADSCs; c‐Heat). c) Relative variations in wound closure ratio of each group over 14 days, compared to day 0 (mean values ± s.d.; sample size = 6, two‐way ANOVA). d) Representative hematoxylin and eosin (H&E), Masson's Trichrome, and immunofluorescence‐stained sections obtained at the skin‐tissue interface between the integrated patch and the skin wound site in each group (Scale bar = 250 µm). e–g) Relative gene expression of fibronectin (e), keratine 14 (f), involucrin (g), and CD31 h) at the skin‐tissue interface in each group was evaluated by qRT‐PCR (Sample size = 6, one‐way ANOVA).

For assessing the therapeutic effects of PTSCL for wound healing, we used an in vivo mouse skin wound model. **Figure** **6**a depicts a photograph and schematics of the wound model. A skin wound (2 cm‐by‐2 cm in size) was formed in the dorsal site of the mouse, followed by a total of eight surgical sutures in each wound edge to prevent skin contracture during wound closure.^[^ ^36^ ^]^ Subsequently, the hADSC‐loaded patch combined with a wireless module was attached to the wound site, covered with a transparent Tegaderm film for reliable fixation, and transferred the subjects into the wireless‐powered cage (Figure 5d) to begin PTSCL therapy. Additionally, to enable a direct comparison with the PTSCL therapy, we included an additional experimental group that received thermal stimulation alone via a wireless Joule‐heating source applied to the skin wound. Figure S30 (Supporting Information) shows this wireless thermal actuator, consisting of 3 × 3 resistors (R = 330 Ω), to deliver the heat to stem cells at a specific temperature ranging from 38 to 39 °C. Animal models were divided into 5 groups, including simple wound dressing encapsulation (i.e., Tegaderm) without hADSCs (Teg), no treatment with hADSCs (c‐NT), LED light stimulation with hADSCs (c‐LED), thermal stimulation with hADSCs (c‐Heat), and both photothermal and light stimulation with hADSCs (PTSCL). The cage antenna wirelessly powered the wireless LED module and thermal actuator for 24 h to stimulate hADSC spheroids into the micro‐hole patch. After allowing photothermally upregulated spheroid delivery for 3 days, the entire modules were removed from the subjects and replaced with Tegaderm for all experimental groups. Regardless of photothermal stimulation, cells were no longer retained in the microholes of the patch after being applied to the wound (Figure S31, Supporting Information). To evaluate cell delivery, spheroids were labeled with DiI and monitored via fluorescence imaging. Following stimulation, the patch was removed, and the delivered cells were found to be localized at the wound site, and the cells remained at the wound site for more than 5 days (Figure S32, Supporting Information). The wound closing area in the whole group was monitored for 2 weeks (0, 7, 10, and 14 days, respectively; Figure 6b). The PTSCL group had the highest wound closure rate across all time periods, with the highest wound closure rate in all groups at 7 days post‐treatment. The c‐LED group showed a little increase in therapeutic effect, but less than that of the PTSCL group in all time periods. On the other hand, the wound closure ratio of the c‐Heat group was similar to the other groups for 7 days, except for the PTSCL group, but was observed to fall behind after 10 days (Figure 6c).

After observing wound closure for 14 days, hematoxylin and eosin (H&E) staining and Masson's Trichrome staining were performed for histological analysis (Figure 6d). Results of H&E staining indicate that the PTSCL group had the thickest dermis. Interestingly, the stratum corneum above the epidermal layer was only observed in the PTSCL group, indicating that the PTSCL therapy had the fastest wound healing rate and that the keratinocytes in the outermost layer of the skin undergo terminal differentiation to form the stratum corneum. Masson's Trichrome staining confirmed collagen deposition in each group. Collagen fibers undergo cross‐linking and organization to enhance the structural integrity of the tissue as well as improve the strength and functionality of recovered skin. We observed that the collagen (stained blue) was spread over the largest area under the dermis layer of the PTSCL group. Following histology analysis, we performed immunohistochemistry to evaluate involucrin and CD31 expression. The expression of integrins in skin wounds can be an indicator of the ongoing differentiation and maturation of keratinocytes, which is essential for proper healing and restoration of skin barrier function. Besides, CD31 is considered a marker of endothelial cells, and its expression indicates the presence of a functional vascular network in the recovered skin. In the PTSCL group, the expression of involucrin was not only prominent in the epidermis layer, but also notably observed in the dermis layer. Whereas, histologically, dermis and epidermis layers of the c‐Heat group exhibited less distinct features than other groups. The least CD31 expression was observed in the recovered skin of the Teg group, while the results of the PTSCL group also exhibited the highest CD31 expression, and the growth of blood vessels was evident in the repaired skin. We also performed qRT‐PCR to assess corresponding gene expressions in the remodeled skin (Figure 6e–h). As shown in Figure 6e, we found that the c‐LED group had the highest fibronectin expression, in contrast to the c‐LED group, but there was no significant difference between c‐LED and PTSCL. For keratin 14 and integrin gene expression, there was a significant increase in the PTSCL group, suggesting a synergistic effect of wound closure (Figure 6f,g). CD31 gene expression was also found to be increased in the PTSCL group, showing similar results to CD31 staining (Figure 6h).

Overall, in vivo experimental results confirmed that the PTSCL therapy had effectively recovered the wound not only by its closure, but also actual restoration as normal skin tissue for maintaining skin barrier function (Table S2, Supporting Information). Interestingly, light therapy via LED irradiation, commonly called PBM, showed quite effective wound closure and maturation of skin. It is known that such LED‐based PBM treatment can promote the migration of cells adjacent to the wound site, containing epithelial cells, endothelial cells, and immune cells.^[^ ^37^ ^]^ In particular, red light LED could strengthen the involucrin expression in keratinocyte.^[^ ^38^ ^]^ Thanks to this therapeutic effect, in vivo results using PBM showed enhanced skin maturation and extracellular matrix (ECM) enrichment. However, due to the low light intensity of LED (less than 2.5 mW·cm^−2^) in the wireless module, it has limited access to the epidermal layer of the skin, identified by relatively smaller CD31 staining and gene expression (Figure 6d,h), making it difficult to deliver therapeutic effects to deeper skin layers below the dermis. In contrast, mild hyperthermia treatment using a wireless thermal actuator showed a lower therapeutic effect on skin maturation, but a higher effect on inducing angiogenesis in remodeled skin tissue. Such localized hyperthermia on dermal skin could induce vasodilation of blood vessels.^[^ ^39^ ^]^ which can increase blood flow to the wound site and increase the supply of oxygen and nutrients, including VEGF, for new blood vessel formation.^[^ ^40^ ^]^ Thus, mild hyperthermia treatment can induce an increase in CD31 expression, compared to the c‐LED group (Figure 6d,h). Our findings suggest that mild hyperthermia is effective in promoting vascular regeneration, while LED‐based PBM with low light intensity demonstrates efficacy in skin maturation (Table S2, Supporting Information). Furthermore, when comparing the therapeutic effects of LED, photothermal, and heat treatments without hADSC loading, it seems that the overall wound closure ratio showed a similar trend, yet staining results, in particular, involucrin and CD31 expression, elicit dramatically reduced therapeutic efficacy without cell loading (Figure S33, Supporting Information). Additionally, vascular stability in newly formed skin tissue and scar formation were observed 14 days after wound closure (28 days after wound formation). The results demonstrated that in the c‐NT group, which received no photothermal stimulation, the regenerated tissue‐maintained blood vessels comparable in density and structure to those in normal, unwounded skin. Notably, in the PTSCL‐treated group, previously shown to exhibit superior angiogenic responses, we observed a significantly greater number of well‐preserved blood vessels with intact lumens and red blood cell (RBC) presence, indicating both structural durability and perfusion capability (Figure S34, Supporting Information). Also, the PTSCL‐treated group displayed collagen organization and staining intensity comparable to that of normal skin. Importantly, there was no evidence of excessive blue staining, which would typically indicate pathological collagen accumulation. Similarly, the c‐NT group also showed no signs of abnormal collagen overproduction. These results suggest that PTSCL therapy does not promote fibrotic scarring and supports the formation of functionally integrated tissue without detrimental ECM remodeling (Figure S34, Supporting Information). Based on these results, we confirmed the potential for a synergistic effect of PTSCL therapy by combining the therapeutic benefits of mild hyperthermia and LED‐based wireless PBM with highly upregulated stem cell spheroid delivery (Table S2), Supporting Information. While the present study demonstrates the pro‐regenerative effects of PTSCL therapy in an acute wound model, this model does not fully replicate the pathological complexity of chronic wounds, such as diabetic ulcers. Acute wound models were intentionally chosen in this proof‐of‐concept study to establish the basic feasibility, biocompatibility, and therapeutic potential of the PTSCL system under well‐controlled conditions. However, to fully evaluate the translational applicability of this strategy, further studies in chronic wound models are warranted.

## 3 Conclusion

The PTSCL therapy introduced here represents a synergistic approach to wound healing with fully wireless optoelectronics and stem cell spheroids, aiming to accelerate the process and enhance therapeutic efficacy. Our patch is designed to be flexible and composed of commonly used biocompatible materials such as PDMS and Au/nanoturf membranes, making it suitable for safe and reproducible applications. Additionally, we have confirmed that the photothermal properties of the patch are well maintained even after multiple rounds of autoclave sterilization, a widely accepted method in clinical settings. This durability further supports the feasibility of mass production and safe deployment in hospital environments. Photothermal stimulation operates at a mild temperature (≈39 °C), which minimizes the risk of thermal damage and enhances compatibility for clinical use. This therapy supports one‐step treatment, involving the formation and delivery of a stem cell spheroid within a skin wound patch for 24 h at the initial stage. Additionally, 630 nm light irradiation of a wireless LED module enhances the therapeutic effect of laden spheroids by inducing mild hyperthermia through the photothermal effect of the Au/Nanturf structure. Notably, each approach of physical modulation, such as mild photothermal‐hyperthermia and light irradiation, also showed elevated therapeutic efficacy, but we observed that the PTSCL treatment has the most improved therapeutic outcomes through the synergistic effect than others, as validated from both in vitro and in vivo experimental results. Importantly, our system leverages physical stimulation without involving chemical modifications or genetic engineering of the cells. This approach allows for easier regulatory integration with existing and approved stem cell therapies, which is highly favorable for clinical translation.

As such, our demonstration suggested an intriguing perspective for applying multiple cell modulation to strengthen the wound healing properties of stem cells without any cytotoxicity, which is due to applying a relatively low intensity of LEDs for an extended period. The proposed scheme has the potential to be expanded to utilize stem cell therapy in regenerative medicine for treating chronic wounds caused by diabetes, as well as other diseases like Parkinson's disease, spinal cord injuries, and cardiovascular disorders. This is because the red LED wavelength we use in this work is flexible in terms of device footprint, and it is even possible to implement microscale devices, or micro‐LEDs, for miniatured implantable stem cell therapy. Furthermore, integrating options for in situ monitoring of wound closure^[^ ^41^, ^42^, ^43^, ^44^ ^]^ would significantly expand the range of clinically relevant applications.

## 4 Experimental Section

### Fabrication of Au/Nanoturf Membrane‐Embedded Skin Wound Patch

A polydimethylsiloxane (PDMS, 10:1 mixing ratio) slab (width: 2.1 cm and length: 2.1 cm) with a protruded microcylindrical array pattern (diameter: 540 µm and height: 50 µm) was prepared, and the top surface of the microcylindrical array in the upper PDMS slab was then temporarily bonded to the bottom glass substrate. Polysilioxane acrylate in the uncured liquid state was spontaneously filled the space (50 µm‐thick) underneath the PDMS slab owing to its capillary flow. After partial curing of PSA with UV flood exposure (MT‐UV‐A31, Minuta Tech, Korea), the PDMS slab was demolded from the substrate, followed by dry etching via a reactive ion etching (RIE) system with argon gas (Ar) to define randomly‐distributed nano‐scale turf structure with micro‐through hole (diameter: 540 µm and thickness: 50 µm) membrane.^[^ ^16^ ^]^ Ti and Au layers were sequentially deposited onto the top surface of the nanoturf membrane by sputtering (Q300T D, Quorum Technologies Inc.) to attain the desired Au thickness. Au‐coated nanoturf (Au/Nanoturf) membrane was peeled off from the glass substrate by a sharp razor blade, then through‐holes of the membrane were positioned into a positively protruded micropillar array (diameter: 500 µm and height: 300 µm), fabricated by SU‐8 photoresist on a silicon wafer. PDMS precursor with a 10:1 mixing ratio was poured on the membrane‐mounted wafer substrate, and then cured in a 70 °C convection oven for 2 h without trapped air bubbles. Finally, the Au/Nanoturf membrane embedded PDMS slab for skin wound patch was gently demolded.

### Optical and Thermal Characterization

The morphology and geometry of the nanoturf membrane before and after Au deposition were measured by scanning electron microscopy (Hitachi S‐4800, Japan) and a digital camera (EOS 60D DSLR, Canon Inc., Japan), respectively. The optical properties were assessed by a UV–vis–NIR Spectrophotometer (Cary 5000, Varian, Australia) with a wavelength ranging from 400 to 1100 nm, whereas the wavelength of the received LED was confirmed using light spectrometry (HR4000, Ocean Optics Inc., USA). Furthermore, the light intensity of the modules comprising a 3 × 3 LED array was examined using an optical power and energy meter (PM100D, THORLABS GmBH, Germany) at an optical wavelength of 630 nm. An IR camera (FLIR T420, FLIR Systems Inc., USA) obtains the temperature distribution of the Au/Nanoturf membrane‐embedded skin wound patch on the anesthetized mouse with LED power ON/OFF for 5 min.

### Fabrication of a Wired LED Module for In Vitro 3D hADSC Spheroid Testing

A 630 nm red LED (SML‐P11UTT86, Rohm semiconductor) was chosen as a light source for this study. Standard soldering process bonded the surface‐mounted components including the LEDs (3‐by‐3) and resistors (100 Ω) in each row onto the pads of a flexible printed circuit board (fPCB) fabricated on a polyimide substrate (Thickness: 25 µm) The encapsulation process of a wired LED module began with pouring 1.5 g of white‐dye mixed PDMS (10 wt.% white dye with PDMS precursor; 10:1 mixing ratio) into petri dish (2 inch in diameter) and curing at 80 °C hot plate for 10 min, yielding a partially cured, sticky PDMS surface. The component‐mounted fPCB was placed onto the surface, then fully covered by an uncured PDMS (2 g). After removing the entrapped air bubbles underneath the fPCB in a vacuum chamber, soft encapsulation layers were thermally cured at an 80 °C oven for 12 h. A mechanical punch with a diameter of 1.5 mm defined the openings for wiring connection with thin copper wires (38 AWG enameled copper, Remington Industries, USA).

### Cell Culture

hADSCs were purchased from Lonza (Bazel, Switzerland). As obtained hADSCs were cultured in Dulbecco's Modified Eagle's Medium (DMEM; Gibco BRL Gaithersburg, MD, USA) supplemented with 10% (v/v) fetal bovine serum (Gibco BRL) and 1% (v/v) penicillin/streptomycin (Gibco BRL). RAW 264.7 cell lines were purchased from ATCC (Cat #: TIB‐71, RRID: CVCL_0493, purchased in March 2023, Manassas, VA, USA) and cultured in DMEM (Gibco BRL) supplemented with 10% (v/v) fetal bovine serum (Gibco BRL) and 1% (v/v) penicillin/streptomycin (Gibco BRL). Both cells were incubated at 37 °C and 5% CO2 saturation. The cell culture media were changed every 2 days. For M1 polarization of RAW 264.7 cells, LPS (1 µg mL^−1^) was added and incubated for 24 h. Both cells within passages 5–7 were used.^[^ ^45^ ^]^ hADSCs and RAW 264.7 cells were confirmed to be negative for HIV‐1, hepatitis B & C, mycoplasma, bacteria, yeast, and fungi.

### Rationale for Cell‐Line Use and Effect on Conclusion

The murine macrophage line RAW 264.7 was employed as a well‐characterized, reproducible model of innate immune activation that exhibits robust and predictable polarization responses to LPS. After inducing an M1‐like state with LPS, CM collected from each experimental group was applied to test whether each group‐derived secretome could drive M1‐to‐M2 phenotypic transition under controlled conditions. Consequently, the findings from this assay support the conclusion that factors released by the respective groups possess the capacity to modulate macrophage phenotype toward an M2‐like state in an inflammatory background.

### In Vitro Cell Testing with 3D hADSC Spheroids

In vitro cell testing with 3D hADSC spheroids began with the preparation of PDMS square wall containers with a width (W), length (L), and height (H) of 2.1, 2.1, and 1 cm, respectively, in a six‐well cell culture plate (Corning Inc., Corning, New York, NY, USA) for hADSC loading and culturing. The lateral dimension of the square pit was determined by the Au/Nanoturf membrane‐embedded skin wound patch (2 cm‐by‐2 cm), minimizing cell loss for 3D spheroid formation. The wound patch was temporarily fixed to a glass substrate (2 cm‐by‐2 cm, 1 mm thick) to avoid deformation in the cell culture medium during the cell culturing process. After placing the patches into each container, ≈2 mL of sterile PBS solution was added to the container, followed by centrifugation (2000 g, 5 min) to completely remove entrapped air bubbles. Next, cells were loaded into 2 mL of cell culture media into the container, followed by centrifugation (400 g, 5 min) to load the cells into each microwell cavity of the skin wound patch (10^6^ cells/patch). The cell culturing plate was incubated for 24 h in a 5% (vol/vol) CO2 incubator at 37 °C. A wired LED module was attached underneath the well‐plate after 24 h incubation, then a precision source/measure unit (B2901A, Agilent Technologies Inc.) provided the required electrical power of 84 mW, yielding a light intensity (≈1.66 mW·cm^−2^) to induce photothermal effect in the LED array. Additional culturing procedures for 0, 1, and 24 h were performed in each experimental group. (for details, see Figures S7 and S10, Supporting Information).

### Live and Dead (FDA/EB)

FDA/EB staining was performed using fluorescein diacetate (FDA, Sigma–Aldrich, St. Louis, MO, USA) and ethidium bromide (EB, Sigma–Aldrich). FDA (green) stains the cytoplasm of viable cells, whereas EB (red) stains the nuclei of nonviable cells. The staining solution was freshly prepared by combining 10 mL of FDA stock solution (1.5 mg mL^−1^ of FDA in dimethyl sulfoxide), 5 mL of EB stock solution (1 mg mL^−1^ of EB in PBS), and 3 mL of PBS. hADSCs were then incubated with the staining solution for 3 min at 37 °C. After staining, the samples were washed twice with PBS and examined using a fluorescence microscope (DFC 3000 G, Leica, Wetzlar, Germany).

### Apoptotic Activity

For apoptotic activity, the spheroids were extracted from the PDMS patch and embedded in optimal cutting temperature (OCT, SciGen Scientific, Gardenas, USA) compound. The OCT block was cut into 10 µm‐thick sections at −22 °C. A terminal deoxynucleotide transferase‐mediated deoxyuridine triphosphate nick‐end labeling (TUNEL) assay was performed using the ApopTag Fluorescent In Situ Apoptosis Detection Kit (Millipore, Bedford, MA, USA) to examine the apoptotic activity of each group. Following 4,6‐diamidino‐2‐phenylindole (DAPI, Vector Laboratories, Burlingame, CA, USA) staining, TUNEL‐positive fluorescence was measured using a fluorescence microscope (DFC 3000G, Leica).

### Flow Cytometry

hADSCs spheroid cultured on the PDMS patch were dissociated by Accutase (Gibco BRL) treatment for 15 min at 37 °C. The hADSCs in each group were centrifuged for 5 min at 1500 rpm. Then, hADSCs were washed twice with cold cell staining buffer (Biolegend, San Diego, CA, USA) and treated with 100 Annexin V binding buffer (Biolegend) at a concentration of 3 × 105 cells/100 µL. After treating with Annexin V binding buffer, the hADSCs were transferred to test tubes. Annexin V (Biolegend) and 7‐aminoactinomycin D (7‐AAD, Biolegend) solution were subsequently added to the hADSC in the test tubes. Annexin V binds to phosphatidylserine on the external leaflet of the plasma membrane of apoptotic cells, while 7‐AAD solution penetrates the nuclear membrane and binds to the DNA of necrotic cells. Next, the test tubes were gently vortexed and incubated for 15 min at room temperature. Additional Annexin V binding buffer (200 µL) was added to the hADSCs in the test tubes, and the hADSCs were analyzed by flow cytometry with a CytoFLEX Flow Cytometer (Beckman Coulter, Brea, CA, USA). Figure S35 (Supporting Information) shows the gating strategy.

### Quantitative Reverse Transcription Polymerase Chain Reaction (qRT‐PCR)

The relative gene expression levels of each experiment were quantified using quantitative reverse transcription polymerase chain reaction (qRT‐PCR). Total ribonucleic acid (RNA) was extracted from the samples using 1 mL TRIzol reagent (Life Technologies, Inc., Carlsbad, CA, USA) and 200 µL chloroform. The lysed samples were centrifuged at 12 000 rpm for 10 min at 4 °C. The RNA pellet was dried after being rinsed with 75% (v/v) ethanol in water. The samples were then dissolved in RNase‐free water. The SsoAdvanceed Universal SYBR Green Supermix kit (Bio‐Rad, Hercules, CA, USA) and CFX Connect real‐time PCR detection system (Bio‐Rad) were used for qRT‐PCR. The primers used for qRT‐PCR are listed in Table S1 (Supporting Information).

### Inhibition of AKT, ERK, and HIF‐1α Signaling Pathways

To inhibit AKT, ERK, and HIF‐1α signaling, the following pharmacological inhibitors were used: MK‐2206 (AKT inhibitor, Sigma–Aldrich), U0126 (ERK inhibitor, Sigma–Aldrich), and SYP‐5 (HIF‐1α inhibitor). Stock solutions of MK‐2206 (1 mM) and SYP‐5 (1 mM, Sigma–Aldrich) were prepared in Dimethyl sulfoxide (DMSO, Sigma–Aldrich), while U0126 was dissolved in DMSO at a concentration of 10 mM. After spheroid formation within the micro‐hole patch platform, photothermal stimulation was applied, during which the inhibitors were concurrently administered at the following working concentrations: MK‐2206 at 1 µM, U0126 at 10 µM, and SYP‐5 at 1 µM.

### Enzyme‐Linked Immunosorbent Assay (ELISA)

To analyze the secretion of paracrine factors in CM from each group, ELISA kits were used for human VEGF, HGF, KGF, and FGF2 (R&D Systems), according to the manufacturer's instructions. The CM was collected after 24 h of treatment with each stimulation. The OD value of each well was measured at 450 nm using a microplate reader (correction at 540 nm; Infinite F50; TECAN, Männedorf, Switzerland).

### Immunofluorescence Staining

For immunofluorescence staining, the stem cell spheroids were extracted from the PDMS patch with a pipette and fixed with a 4% formaldehyde solution. After fixation, spheroids embedded in OCT compound were cut into 10 µm‐thick sections at −22 °C. Subsequently, immunocytochemistry for HSF1 (ab52757, 1:250, Abcam, Cambridge, MA, USA), HSP70 (ab181606, 1:50, Abcam), and Hif‐1α (ab2185, 1:400, Abcam) were performed. Positive fluorescence signals were visualized using fluorescein isothiocyanate‐conjugated secondary antibodies (Jackson ImmunoResearch Inc., West Grove, PA, USA). The cells were counterstained with DAPI and phalloidin (Sigma–Aldrich) and evaluated under a fluorescence microscope (Leica).

### Dot Blot Assays

Human angiogenesis (ARY007, R & D Systems, Minneapolis, MN, USA) and cytokine array profiling (ARY005B, R & D systems) were conducted following the manufacturer's protocols.

### HUVEC Tube Formation Assay

Tube formation of HUVECs was conducted using an Angiogenesis assay kit in vitro (ab204276; Abcam) following the manufacturer's protocols. The CM extracted from each group was mixed with HUVEC medium at a 1:1 ratio and then used to treat HUVECs cultured on pre‐coated Matrigel.

### Fibroblast Migration Assay

Fibroblast migration assays were performed using 6‐well plates. Briefly, 1 × 10^5^ cells were added to each well and scraped 24 h after seeding. After scraping, cells were washed once with PBS and changed to serum‐free media. Each of the groups was treated with CM extracted from each group. The number of cells that migrated into the scraped area was imaged using brightfield microscopy connected to an optical microscopy (CKX53, Olympus, Tokyo, Japan). The cell migration area in the captured images was calculated using Photoshop CC (Adobe Systems, San Jose, CA, USA).

### Electromagnetic Simulation of a Wireless LED Module

An 8‐turn coil antenna (width, gap: 0.4 mm) was designed and simulated using RF Pro in Advanced Design System (ADS, Keysight Technologies) and commercially fabricated on both sides of a 130 µm‐thick fPCB (Dielectric constant: 3.3–3.5, PCBWay, China). The self‐resonance characteristics (self‐resonance frequency; $f_{SR}=\frac{1}{2\pi \sqrt{LC}}$ and quality factor; $Q=\frac{Im(Z(1,1))}{Re(Z(1,1))}$) of the as‐fabricated coil antenna was measured by a vector network analyzer (ENA E5063A, Keysight Technologies, USA) and was utilized to obtain the value of the tuning capacitor to match the resonance frequency of the patch to 13.56 MHz.

### Fabrication of Wireless LED and Thermal Actuator Module for In Vivo Testing

Electronic components, including the LEDs (SMLP11UTT86, Rohm Semiconductor), ceramic capacitors (GRM series, Murata Electronics), chip resistors (RC series, YAGEO), Zener diode (BZX884‐B12, Nexperia Inc.), and Schottky diodes (BAS40XY, Nexperia Inc.), were mounted onto the pads of the designed fPCB by standard reflow soldering technique. The rectifier circuit consists of a full‐wave bridge followed by an RC filter to obtain a regulated DC voltage corresponding to the transmitted power. Prior to feeding the harvested power directly to the linear regulator (S‐812C60BPI‐C5OTFU, ABLIC Inc.), a Zener diode was connected. The regulated output voltage (6 V) is fed to the 3 × 3 LED array along with the resistors (R = 100 Ω). The wireless thermal actuator module was prepared by just replacing the LEDs with resistors (R = 330 Ω). The encapsulation process of wireless LED and thermal actuator modules was the same as that of a wired LED module.

### Fabrication of the Engineered Animal Cage

The commercially available acrylic box was purchased with a length, width, and height of 21, 15, and 13 cm, respectively. The transmission loop antenna comprising 20 AWG wire (Remington Industries) starts at the bottom plane of the cage with a length and width of 13 and 6 cm, respectively, and then the loop surrounds the cage with twice turns at heights of 2.5 and 6.5 cm, respectively. To create uniform in‐plane coverage of magnetic resonant coupling at the specific frequency (13.56 MHz), each corner of the intertwined loop is located apart from 2 cm lower and higher along the z‐axis, respectively (See Figure S26, Supporting Information).

### Performance Evaluation of the Wireless System

The wireless power transmitter consists of a NeuroLux system (NeuroLux, USA) comprising a laptop with a graphic user interface to control the power, a power generator, and a tuning circuit along with a custom‐designed cage. The harvested power for different input powers at the transmitting side was evaluated at 4.5 cm above the base of the cage with different loading conditions. The light intensity output was examined by an optical power and energy meter (PM100D, THORLABS GmBH, Germany) for different vertical heights by placing the wireless module on a 5 × 7 custom‐made square array matrix (lateral dimension of one unit cell; 3 cm‐by‐3 cm) formed within the cage space, whereas the angular misalignment was evaluated at the center of the cage. For in vivo trajectory measurement, the individual mouse with and without the patch was placed on an open acryl cage (length: 38 cm, width: 22 cm, height: 18 cm) and a video was recorded for 10 min each. Furthermore, an open‐source platform (idtrackerai) was utilized to trace the trajectory data and the total distance covered.^[^ ^46^ ^]^

### In Vivo Wound Closure Testing

Eight‐week‐old female athymic mice (20–25 g body weight, Orient, Seoul, Korea) were anesthetized using 200 µL xylazine (20 mg kg^−1^) and ketamine (100 mg kg^−1^) diluted in normal saline solution. The skin wound area was marked using a 2 cm × 2 cm square‐shaped stamp on the back of each mouse. The epidermis, dermis, and stratum corneum in the marked areas were surgically removed. After skin removal, eight 6‐0 sutures (AILEE Co., Busan, Korea) were placed at the border of each wound to prevent wound collapse by skin contracture.^[^ ^36^, ^47^ ^]^ Immediately after skin wound modelling, the mice were subdivided into eight groups: the No treatment (Teg‐NT, wounds covered with the commercial skin dressing Tegaderm (3 m Healthcare, St. Paul, MN, USA)); LED (PDMS patch + wireless LED module + Tegaderm); PT (Au/Nanoturf membrane‐embedded skin wound patch + wireless LED module + Tegaderm); Heat (Au/Nanoturf membrane‐embedded skin wound patch + wireless heater module + Tegaderm); c‐NT (Au/Nanoturf membrane‐embedded skin wound patch + stem cell spheroid + Tegaderm); c‐LED (PDMS patch + stem cell spheroid + wireless LED module + Tegaderm); c‐PTSCL (Au/Nanoturf membrane‐embedded skin wound patch + stem cell spheroid + wireless LED module + Tegaderm); and c‐Heat (Au/Nanoturf membrane‐embedded skin wound patch + stem cell spheroid + wireless heater module + Tegaderm). For the stem cell spheroid‐loaded group, 10^6^ cells were delivered to the wound area consistent with the in vitro study, and patches were thoroughly rinsed with PBS prior to in vivo application to eliminate residual culture medium and were maintained in PBS until immediate delivery to the wound site after skin excision. The NT group served as the control group. All animals received care according to the guidelines for the care and use of laboratory animals of Sungkyunkwan University (SKKUIACUC2022‐03‐08‐1, May 2022).

### Stem Cell Spheroid Diameter Distribution Analysis

Image analysis was performed for cell diameter distribution. After the spheroid formed in the Au/Nanoturf membrane‐embedded skin wound patch, images of the stem cell spheroid were snapped in the patch with a microscope. Individual stem cell spheroid diameter was measured with Image J (National Institutes of Health, Bethesda, MD, USA).

### Western Blot Analysis

Samples were lysed in radio‐immunoprecipitation assay (RIPA) buffer (Rockland Immunochemicals Inc., Limerick, PA, USA) for western blot analysis. Protein extract was produced from the supernatant following centrifugation at 10 000 g for 10 min. Protein concentrations were calculated using a Bicinchoninic Acid Assay (BCA assay, Pierce Biotechnology, Rockford, IL, USA). Equal amounts of protein from each sample were combined with a sample buffer and electrophoresed on a 10% (v/v) resolving gel using sodium dodecyl sulfate polyacrylamide gel (SDS‐PAGE). The separated proteins were transferred to immune‐blot Polyvinylidene difluoride (PVDF) membrane (Bio‐Rad). The membranes were blocked with 5% (w/v) skim milk in Tris‐buffered saline (TBS; 50 mM Tris–HCl (pH 7.5), 150 mM NaCl, 2.5 mM KCl) and incubated for 1 h at 25 °C. Then, the membranes were probed overnight at 4 °C with antibodies against glyceraldehyde 3‐phosphate dehydrogenase (GAPDH) (Abcam, ab9485, 1:2000), BAX (Cell Signaling, CS5023, 1:1000, Danvers, MA, USA), BCL‐2 (Abcam, ab182858, 1:2000), p‐ERK1/2 (Cell Signaling, CS9101, 1:1000), ERK1/2 (Cell Signaling, CS9102, 1:1000), p‐AKT (Cell Signaling, CS4060, 1:1000), AKT (Cell Signaling, CS4691, 1:1000). Thereafter, the membranes were incubated with horseradish peroxidase‐conjugated secondary antibody (R&D Systems, HAF017 for GAPDH with 1:2000, for BAX, BCL‐2, p‐ERK1/2, ERK1/2, p‐AKT, and AKT with 1:1000) for 1 h at 25 °C, followed by addition of ECL reagent (TransLab, Daejeon, Korea).

### Histology

Impaired skin tissue specimens retrieved 14 days post‐treatment were fixed with a 4% formaldehyde solution and were embedded in OCT compound (SciGen Scientific). Next, 10 µm sections obtained from the specimens were stained with hematoxylin and eosin (H&E) or Masson's trichrome to assess re‐epithelization.

### Immunohistochemistry

For immunohistochemical staining, the samples embedded in OCT compound were cut into 10 µm‐thick sections at −22 °C. To stain the epidermis of the impaired skin tissue, the sections were immunofluorescence‐stained with antibodies against involucrin (Biolegend, 924401, 1:1000). To stain the vessels in impaired skin tissue, the sections were immunofluorescence‐stained with CD31 antibody (Abcam, ab28364, 1:50). Involucrin and CD31 signals were visualized with fluorescein isothiocyanate‐conjugated secondary antibodies (Jackson Immuno Research Laboratories, West Grove, PA). The sections were counterstained with DAPI and were examined via fluorescence microscopy (DFC 3000 G, Leica).

### DiI‐Labeled In Vivo Imaging

hADSCs cultured on the cell culture plate were treated with DiI (Sigma–Aldrich, Burlington, MA, USA) mixed with DMEM at a ratio of 1:200 for 2 h and then washed twice with PBS. The cells were washed three times with PBS containing 1% FBS to remove excess cell labeling agent. Labeled hADSCs were cultured for 24 h in a patch and treated to the mouse wound areas by PTSCL. Fluorescence and quantitative photon means were analyzed with a Lago‐x system (Spectral Instruments Imaging, Tucson, AZ, USA).

### In Vivo Wound Imaging and Wound Closure Quantification

The macroscopic wound area was quantified by capturing photographs taken at various time points. The wound margin was traced, and the pixel area related to the margin was calculated using a ruler. The location of the healed wound margin was defined as the grossly visible margin of epithelial migration toward the center of the wound and over the granulation tissue bed. The wound ratio was calculated as the percentage of the initial wound area: [1‐(wound area at each time point)/(initial wound area)] × 100%. Morphometric analysis of digital images was performed using Photoshop CC (Adobe Systems, CA, USA).

### Statistical Analysis

All data were presented as mean ± SD. The statistical analysis was performed using GraphPad Prism (GraphPad Software, San Diego, CA, USA). To determine statistical significance, an unpaired Student's *t*‐test was performed to compare two experimental groups, and an ordinary one‐way ANOVA was performed for the three experimental groups. Statistical significance was considered when the *p*‐value was less than 0.05 or 0.01.

## Conflict of Interest

The authors declare no conflict of interest.

## Supporting information



Supporting Information

## Data Availability

The data that support the findings of this study are available from the corresponding author upon reasonable request.
