# Supplementary material for: Gold Nanoturf‐Mediated Wireless Photothermal Upregulation of Human Adipose‐Derived Stem Cell Spheroids for Synergistic Skin‐Wound Closure
Source: Adv Sci (Weinh). 2025 Oct 14;12(47):e15490. doi: 10.1002/advs.202515490 (PMC12713014; doi:10.1002/advs.202515490)
Supplement: Supplementary file 1 — Supporting Information [file ADVS-12-e15490-s001.docx]

Supporting Information

Gold Nanoturf-Mediated Wireless Photothermal Upregulation of Human Adipose-Derived Stem Cell Spheroids for Synergistic Skin-Wound Closure

*Jong Uk Kim*^1,2,†^*, Jiyu Hyun*^1,†^*, Gyan Raj Koirala*^1,2,†^*, Jiwon Kim*^1^*, Sung-Won Kim*^1^*, Gwang-Bum Im*^1^*, Yeong Hwan Kim*^1^*, Young Gil Kim*^1^*, Dong-Hyun Lee*^1^*, Hyun Su Park*^1^*, Young-Ju Jang*^1^*, Young Jin Jo*^1^*, Chanho Jeong*^1^*, Arpan Koirala*^3^*, Janghoon Joo*^4^*, Sang Min Won*^4^*, Suk Ho Bhang*^1,^**, and Tae-il Kim*^1,2,^*

*^1^School of Chemical Engineering, Sungkyunkwan University (SKKU), Suwon 16419, Republic of Korea*

*^2^Biomedical Institute for Convergence at SKKU (BICS), Sungkyunkwan University (SKKU), Suwon, 16419, Republic of Korea*

*^3^Department of Computer Science and Engineering, Kathmandu University, Dhulikhel, Kavre, P.O. Box 6250, Nepal.*

*^4^Department of Electrical and Computer Engineering, Sungkyunkwan University (SKKU), Suwon, 16419, Republic of Korea*

† These authors contributed equally to this work.

*** Authors to whom any correspondence should be addressed.

Suk Ho Bhang, Ph.D., E-mail: sukhobhang@skku.edu, Tel: +82-31-290-7242

Tae-il Kim, Ph.D., E-mail: taeilkim@skku.edu, Tel: +82-31-290-7312

**This PDF file includes:**

Supplementary Figures S1 to S35

Supplementary Table S1 and S2

**Supplementary Figures**


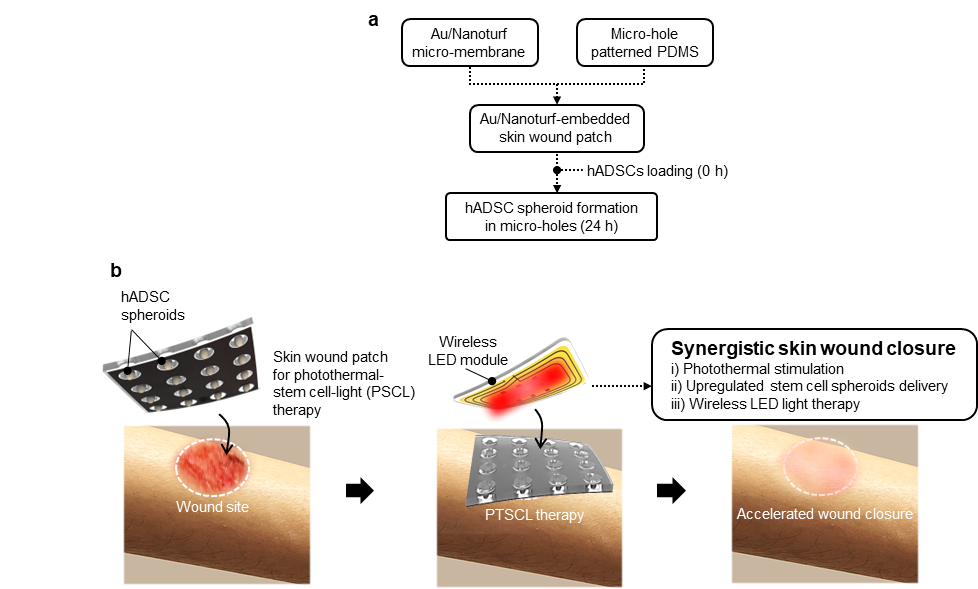


Figure S1. Process flow diagrams for synergistic photothermal-stem cell-light (PTSCL) therapy.

(a) Schematic illustration of the process for forming human adipose-derived stem cell (hADSC) spheroids within an Au/Nanoturf-embedded PDMS patch featuring micro-hole structures. (b) Overview of the proposed PTSCL therapy, integrating mild photothermal stimulation, delivery of photothermally activated hADSC spheroids, and wireless 630 nm LED light therapy for synergistic enhancement of skin wound healing.


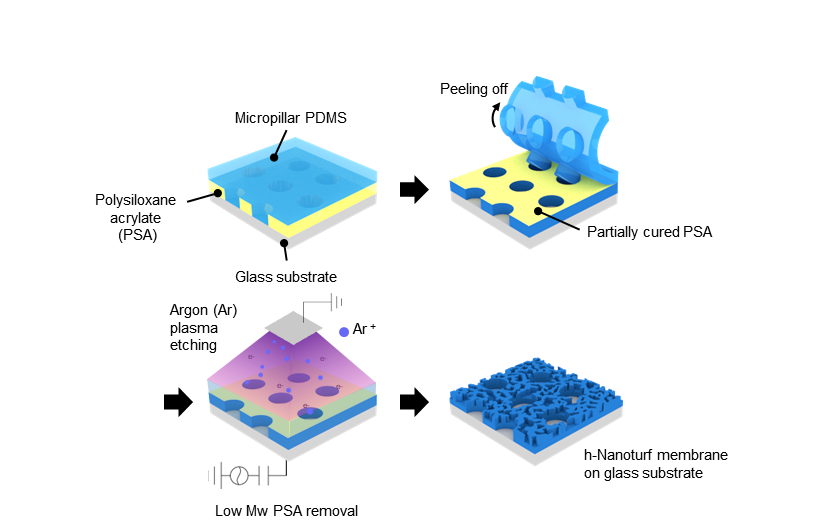


Figure S2. Schematic illustration for fabrication steps of the nanoturf membrane on a glass substrate.


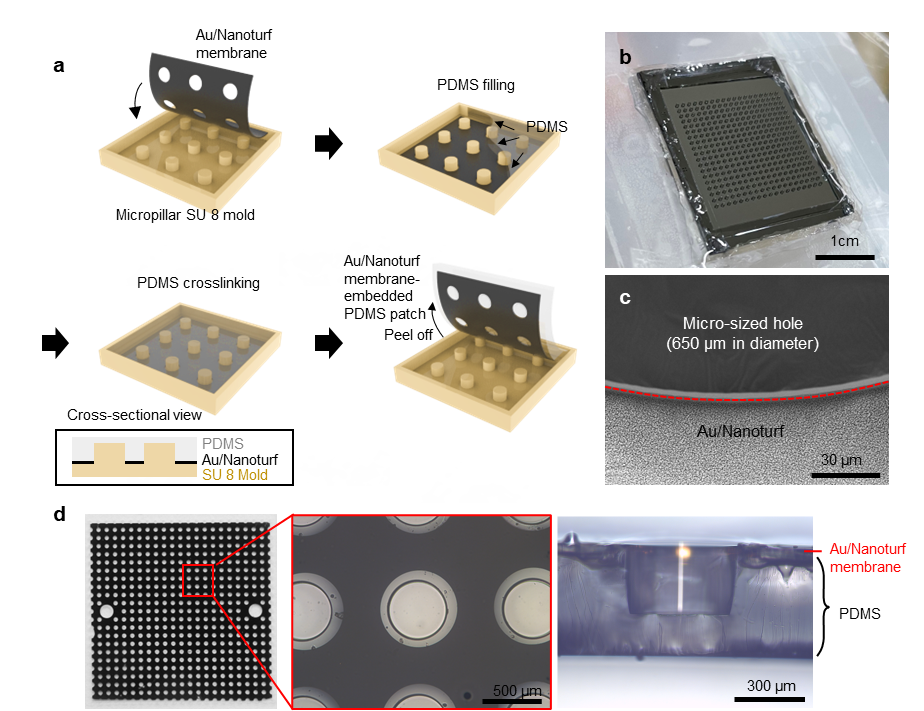


Figure S3. Fabrication processes of Au/Nanoturf-membrane embedded skin wound patch.

(a) Schematic illustrations depicting the assembly steps of the Au/Nanoturf-membrane with SU-8 micropillar structures (500 µm in diameter) on a Si wafer substrate to create a skin wound patch. Bottom inset shows the cross-sectional view during the PDMS crosslinking step. (b) Photograph of the SU-8 micropillar structures on the Si wafer substrate. (c) SEM image showing Au/Nanoturf membrane with micro-sized through hole (600 μm in diameter). (d) Photograph and OM images showing an Au/Nanoturf-membrane embedded skin wound patch. The diameter, height and thickness of the microhole PDMS (center and right) are 500, 300, and 500 µm, respectively.


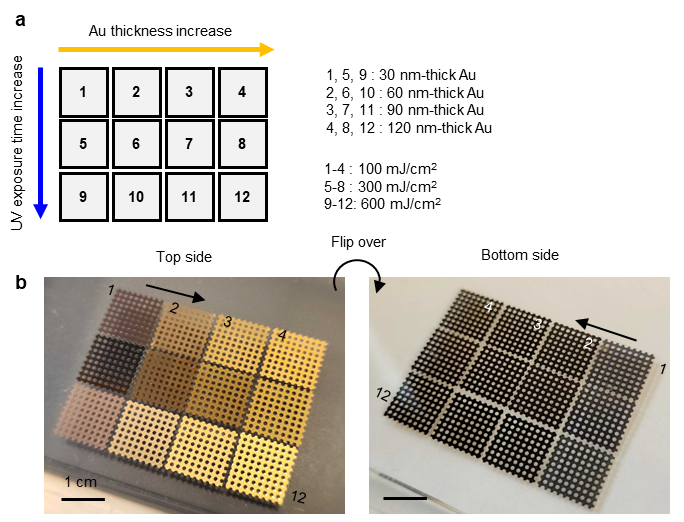


Figure S4. Optical properties of the bilateral surfaces of the nanoturf structure according to a thickness of Au deposition and dosage of UV exposure, respectively.

(a) Schematics describing nanoturf membranes with different fabrication conditions. The thickness of Au deposition and dosage of UV exposure vary from 30 nm to 120 nm and from 100 mJ·cm^-2^ to 600 mJ·cm^-2^, respectively. (b) Photographs showing the top and bottom views of h-Nanoturf samples. Scale bars are 1 cm.


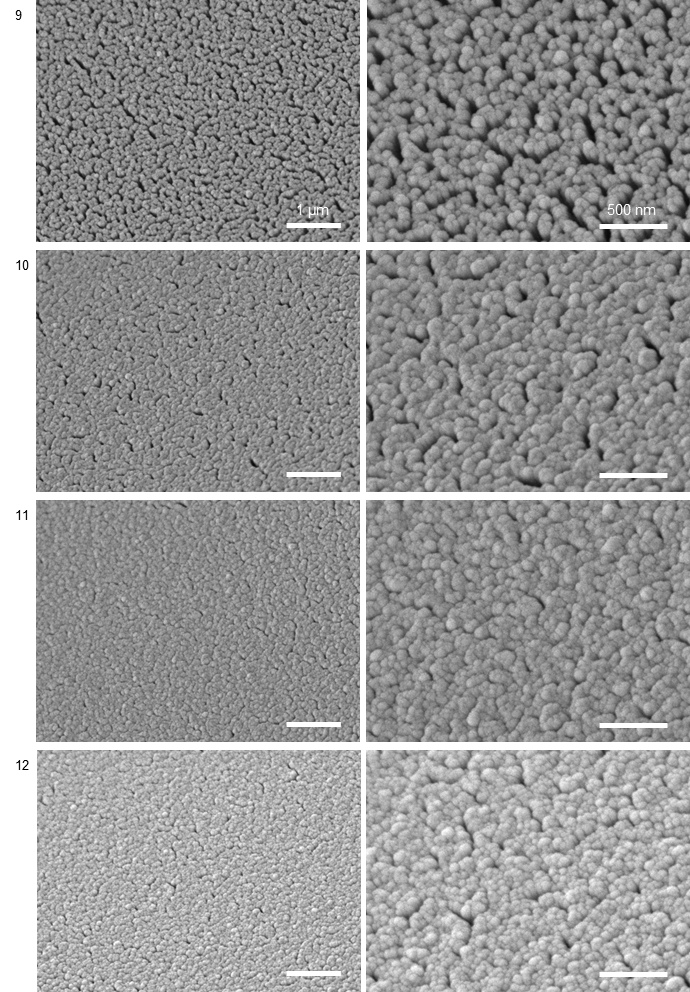


Figure S5. Representative SEM images of nanoturf samples (#9, #10, #11, and #12).

With increasing Au thickness, the top surface openings of the nanoturf structure are gradually clogged, yielding enhanced light reflectance.


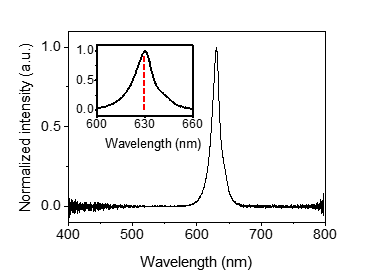


Figure S6. Spectral profiles of the red light-emitting diode (LED) light.


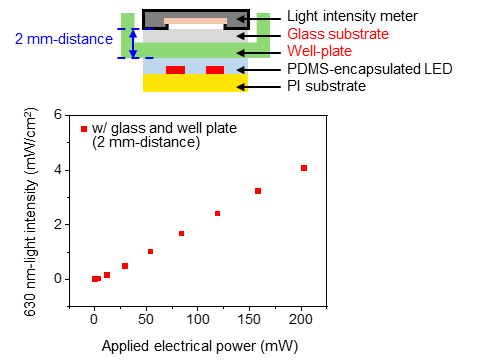


Figure S7. Variation in 630nm-light intensity of the LED light as a function of applied electrical power.

Top inset shows the experimental setup to estimate the 630 nm-light intensity for *in vitro* cell experiment.


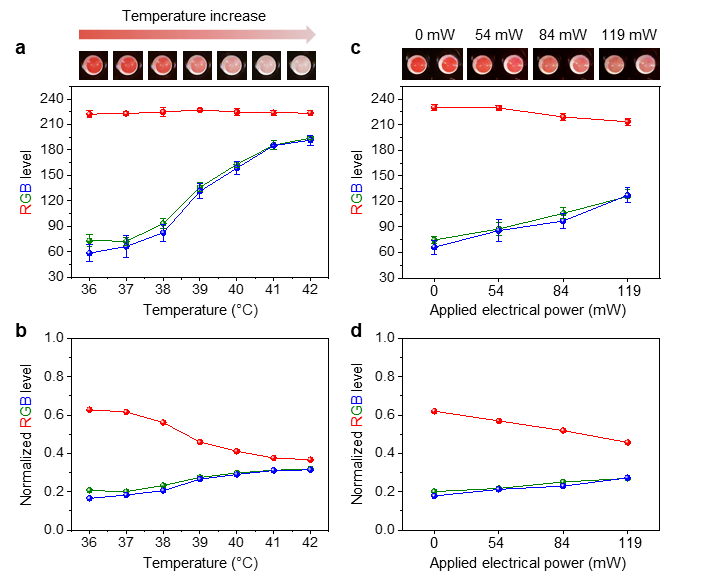


Figure S8. Estimated temperature in micro-holes of the skin wound patch using thermochromic pigment as a function of applied electrical power in the LED module.

(a, b) Extracted (a) and normalized (b) RGB level for reference with response to the temperature elevation. Top inset images display the color change with increasing the temperature. (c, d) Extracted (c) and normalized (d) RGB level with response to the applied electrical power of LED module.


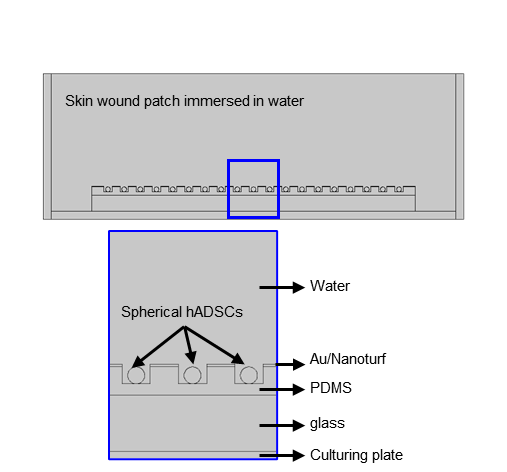


Figure S9. Geometric parameter for finite element analysis (FEA) simulation.


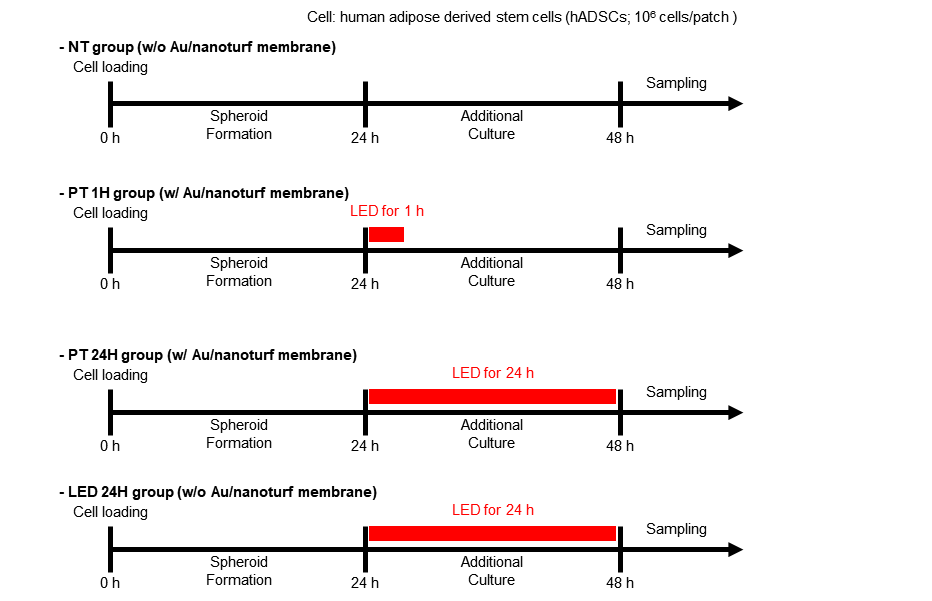


Figure S10. Time sequences of each group for *in vitro* cell testing with 3D hADSC spheroids.


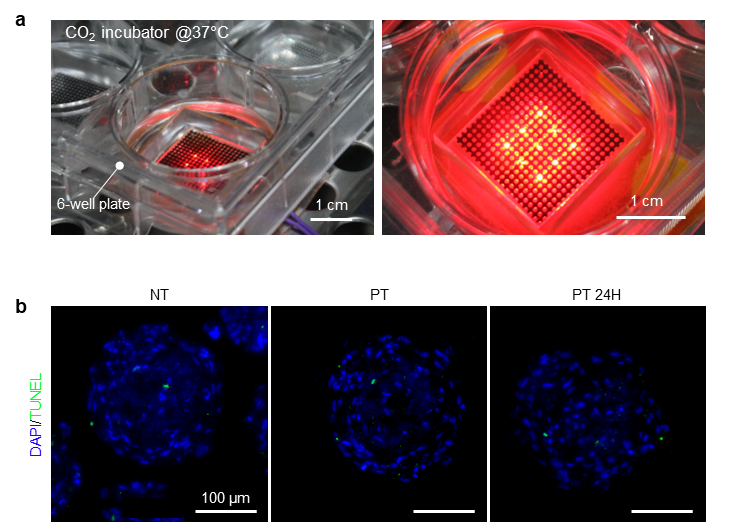


Figure S11. *In vitro* testing of photothermally upregulated hADSCs spheroids loaded in the skin wound patch.

(a) Photographs of the spheroid-loaded patch in CO_2_ incubator at 37 °C. Scale bar is 1 cm. (b) Representative results of the apoptotic activity of a hADSCs spheroid in each group evaluated with the terminal deoxynucleotidyl transferase-mediated dUTP nick end labeling (TUNEL) assay. Apoptotic cells are stained green. Scale bar is 100 μm.


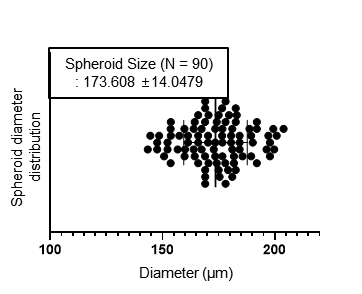


Figure S12. Size distribution (diameter) of hADSCs spheroids formed in the skin wound patch.


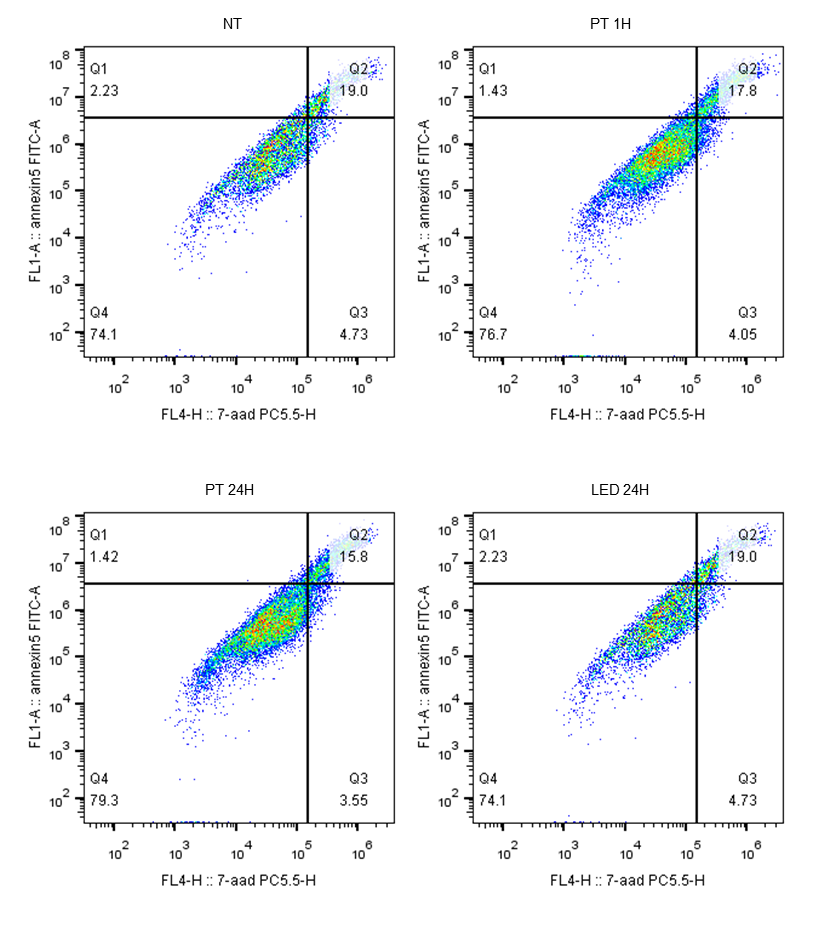


Figure S13. Result of flow cytometry in each group stained with Annexin V and 7-AAD.


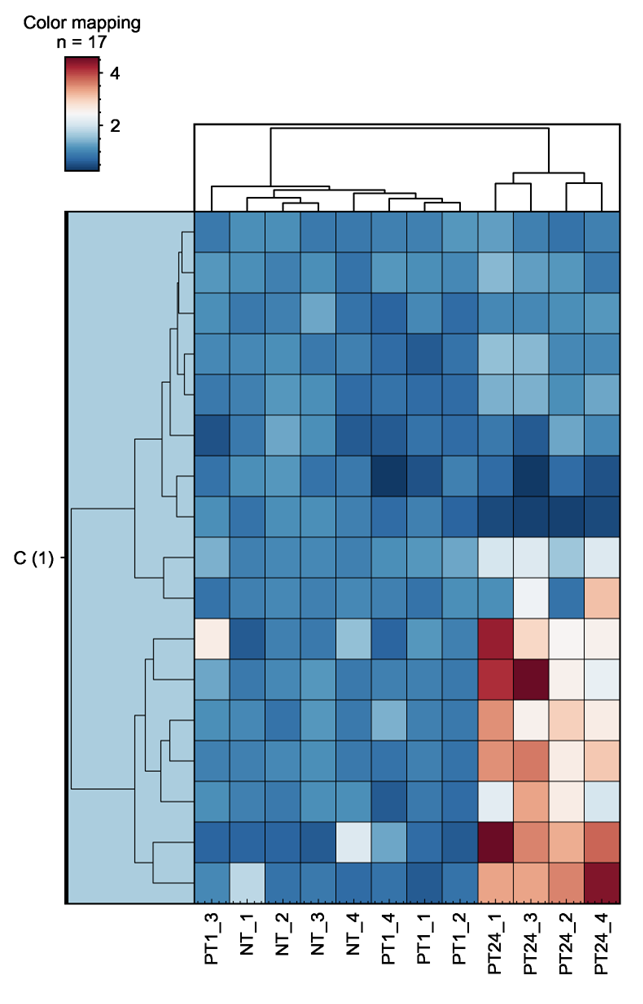


Figure S14. Hierarchical clustering heatmap of gene expression in each group.


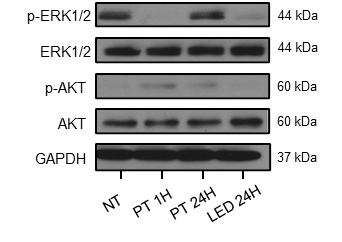


Figure S15. Western blot analysis of ERK and AKT pathway related markers in each group.


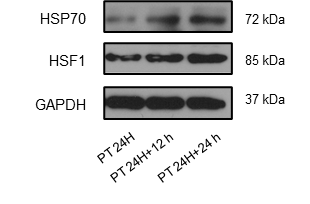


Figure S16. Western blot analysis of heat shock protein expression over 24 hours after photothermal stimulation.


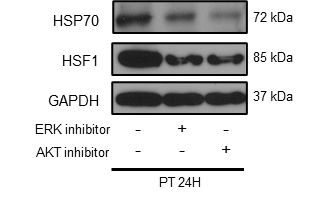


Figure S17. Western blot analysis of HSP expression after inhibition of ERK or AKT pathway.


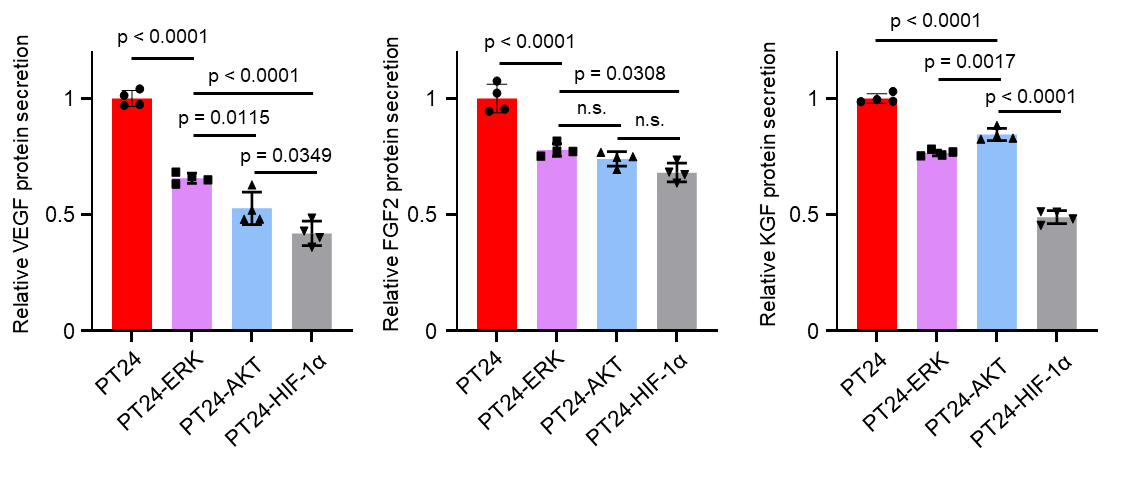


Figure S18. Relative VEGF, FGF2, and KGF secretion in PT 24H, ERK pathway inhibitor treated PT 24H (PT24-ERK), AKT pathway inhibitor treated PT 24H (PT24-AKT), and Hif-1α inhibitor treated PT 24H (PT24-Hif-1α).


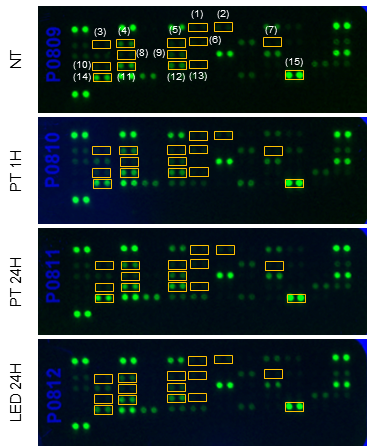


Figure S19. Angiogenesis array dot blot image of each group.


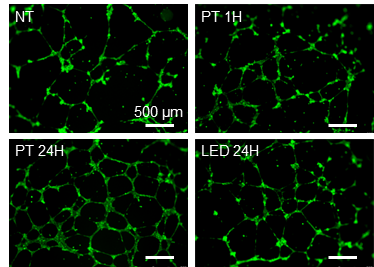


Figure S20. Results of tube formation of human umbilical vein endothelial cells treated with CM in each group. Scale bar is 500 µm.

**
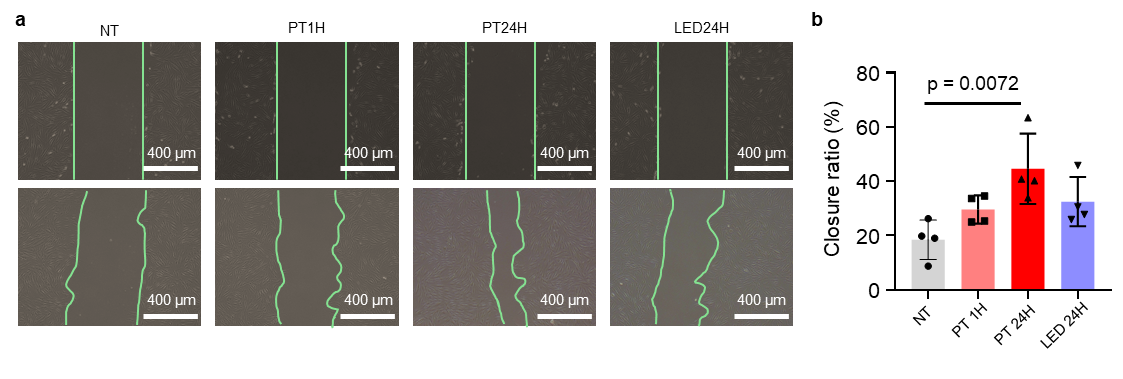
**

Figure S21. Representative image of fibroblast migration assay and quantification of wound closed area in each group.


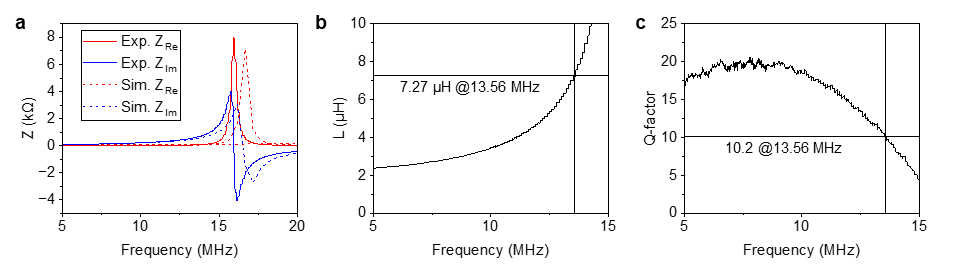


Figure S22. Characteristics of the double-layered coils with 4 turns (Rx coil) obtained from both simulation (Sim.) and the experiment (Exp.).

(**a**) Impedance (Z) spectrum. (**b**) Inductance (L). (**C**) *Q*-factor.


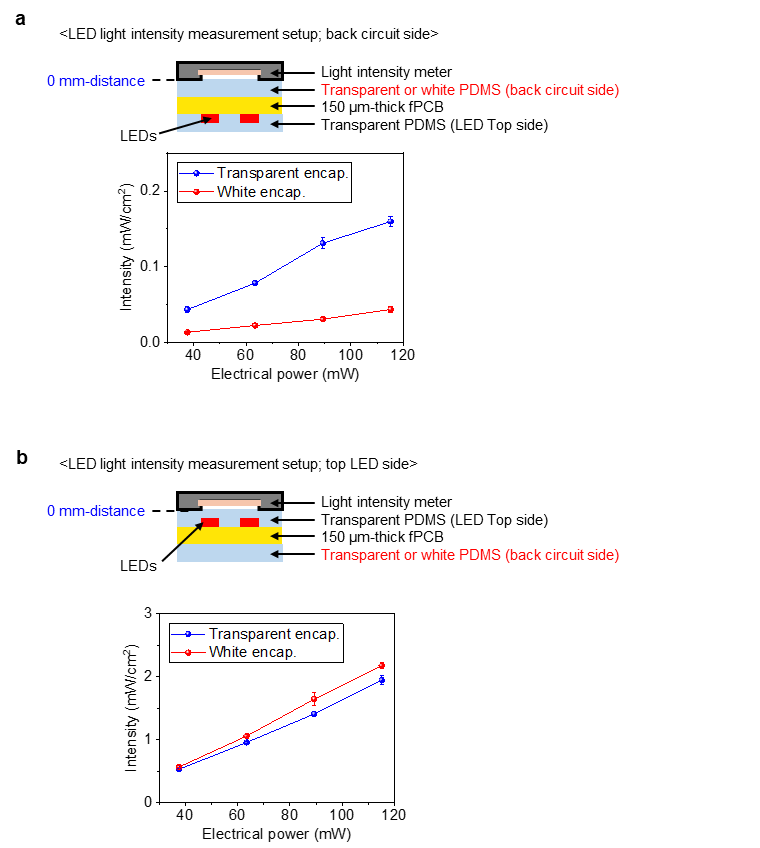


Figure S23. Variation in LED light intensity according to the back circuit side encapsulation layer.

(**a**, **b**) LED light intensity on the back circuit side (**a**) and top LED side (**b**), respectively. Inset schematics illustrate the intensity measurement setup. Transparent (blue) or white (red) PDMS layers were applied to encapsulate the back circuit side, respectively.


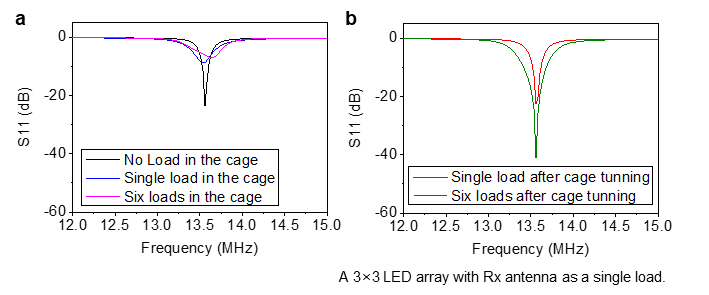


Figure S24. Variation in resonant frequency of the cage antenna with the different number of loads. Here, the load refers to the number of wireless LED module placed within the animal cage.

(**a**, **b**) Resonant frequency data with no load (black), single load (blue/red), six loads (magenta/green) in the cage before (**a**)/after tuning (**b**).


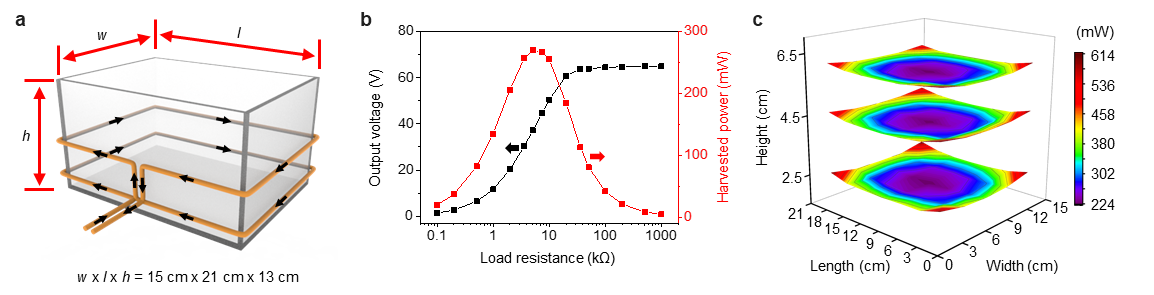


Figure S25. Experimental results for wireless power transmission from a cage antenna with conventional double-loop design.

(**a**) Schematic illustration of cage antennas with conventional design. (**b**) Variations in harvested output voltage and power as a function of load resistance. (**c**) Output power distribution as a function of in-plane position at three different heights (2.5 cm, 4.5 cm, and 6.5 cm) for the case of a double-loop antenna with turns at heights of 2.5 and 6.5 cm. A load resistance is 5.1 kΩ.


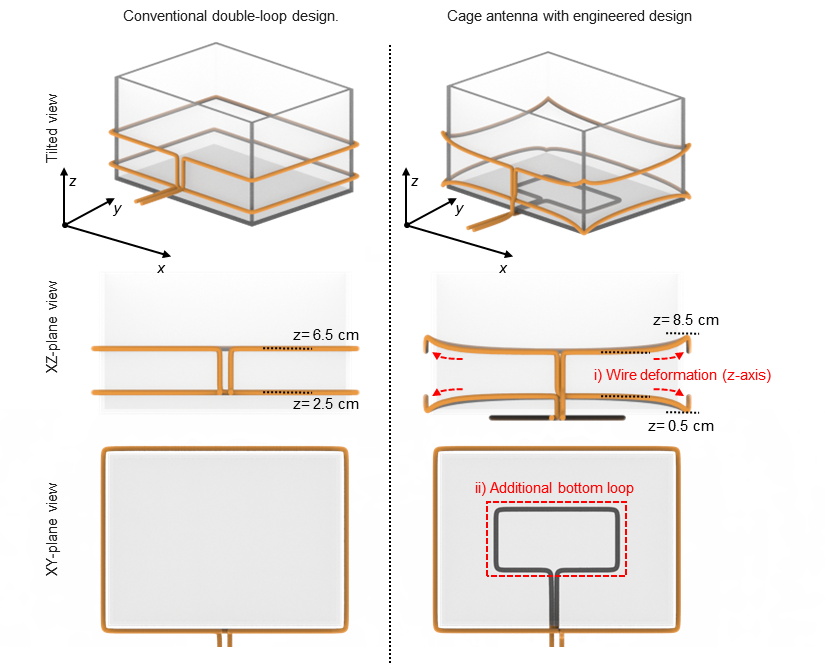


Figure S26. Schematic illustrations comparing a conventional double-loop cage antenna with the engineered design.

Main changes are i) wire deformation at each corner of the double loop antenna with 2 cm lower and higher along the z-axis, respectively, and ii) additional bottom loop to achieve uniform power distribution within the cage.


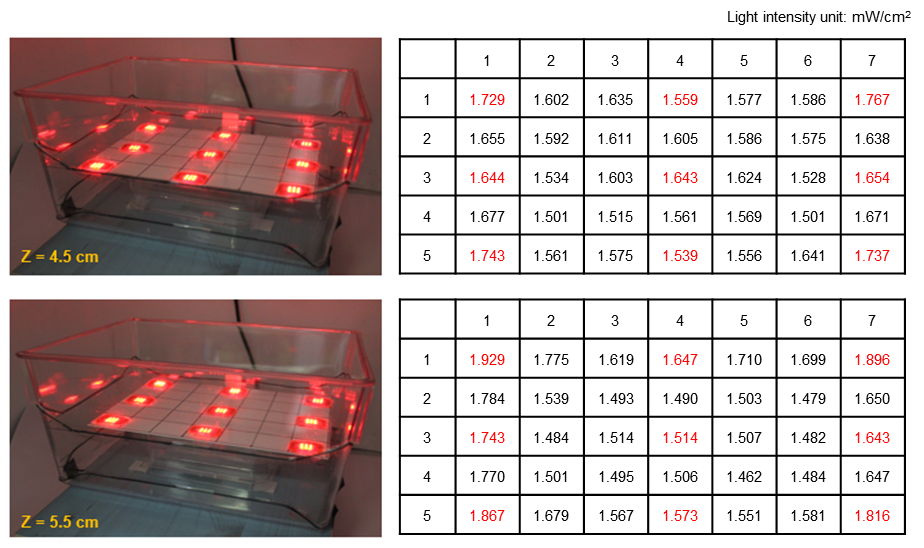


Figure S27. LED light intensity distribution as a function of in-plane position at two different heights (top; *h* = 4.5 cm and bottom; *h* = 5.5 cm).

The numbers of rows and columns are 5 and 7, respectively. Each cell dimension is 3 cm-by-3 cm.


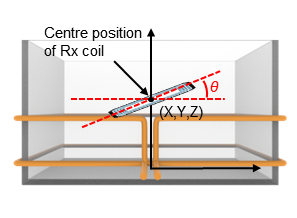


Figure S28. Schematic illustration showing LED intensity measurement setup at the center position with different angular misalignments (*θ*).

**
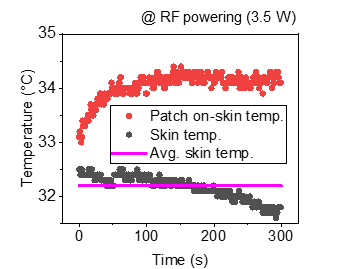
**

Figure S29. Estimated patch on-skin and skin temperatures with RF powering.

Red and black dots are patch on-skin and skin temperature with RF powering (3.5 W), respectively. Magenta line is average skin temperature (32.2 °C) with RF powering.


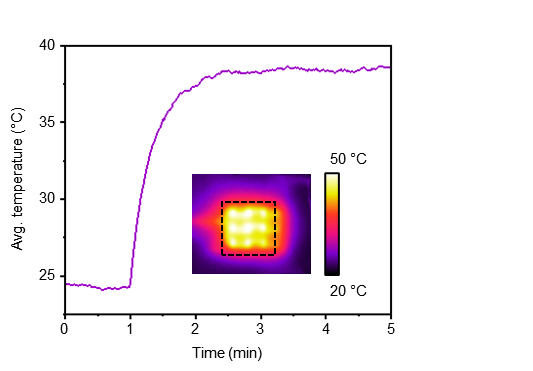


Figure S30. Temperature distribution of the wireless thermal actuator.


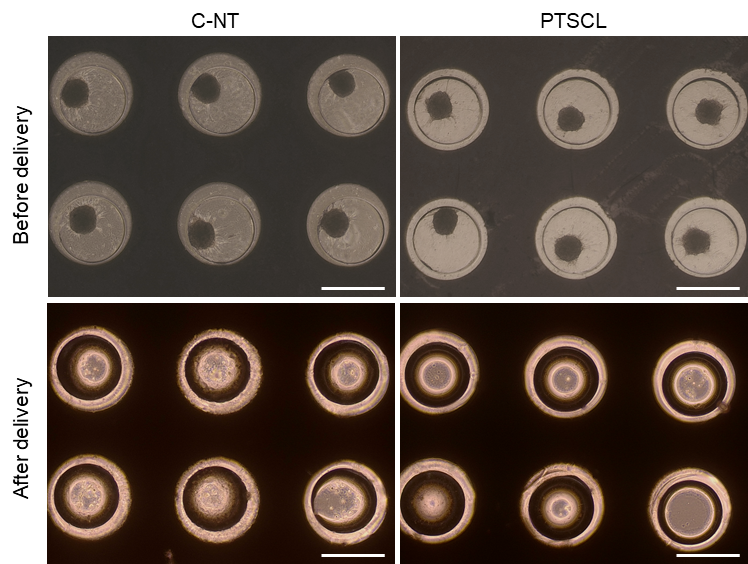


Figure S31. Optical image of patch before and after delivery in mouse skin wound (Scale bar = 500 μm)


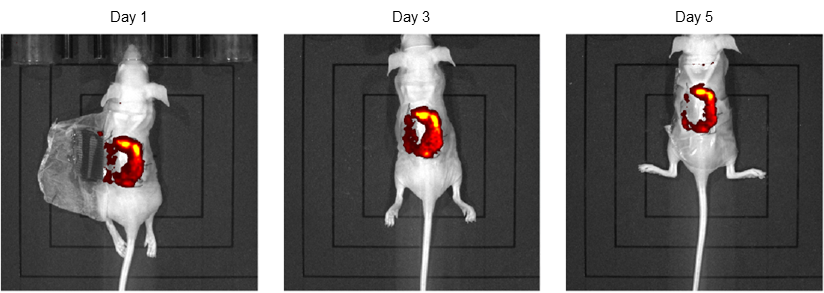


Figure S32. Fluorescent image of DiI-labeled hADSC after delivery with patch.


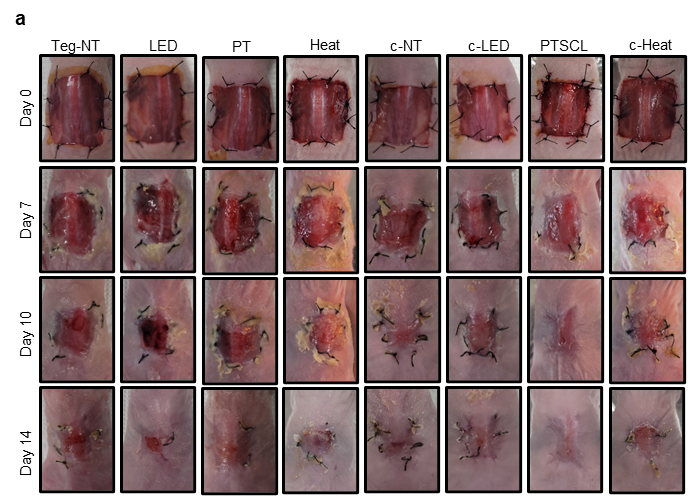


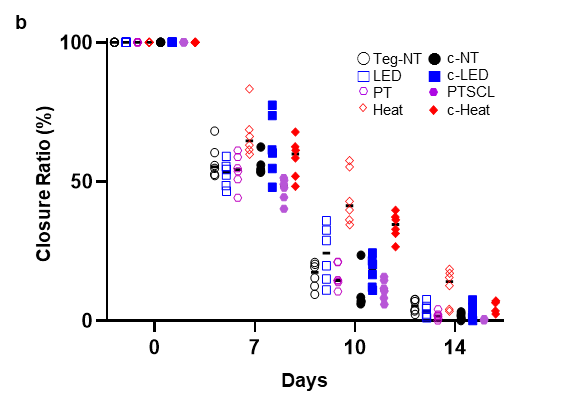


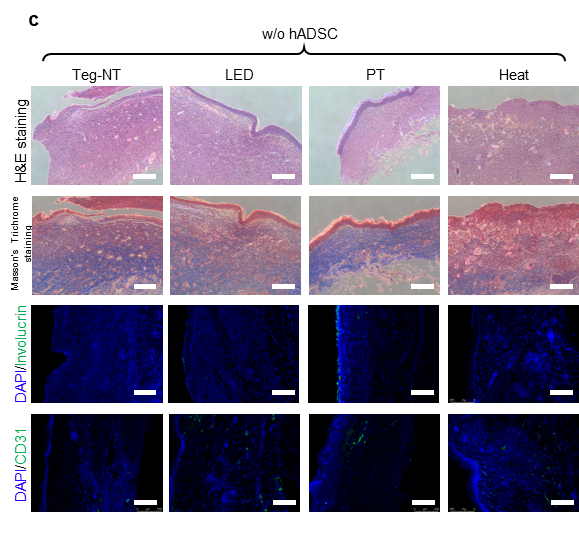

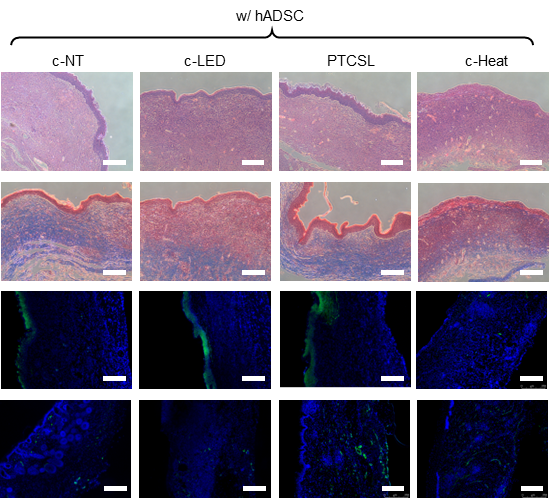


Figure S33. *In vivo* wound healing efficacy of the whole group.

(**a**) Representative photographs of sequential skin wound closure for 14 days in each group (no treatment without hADSCs; Teg-NT, LED treatment; LED, photothermal treatment; PT, thermal treatment; Heat, with hADSCs; c-NT, LED treatment with hADSCs; c-LED, both photothermal and light stimulation treatment with hADSCs; PTSCL, and thermal treatment with hADSCs; c-Heat). (**b**) Relative variations in wound closure ratio of each group over 14 days, compared to day 0 (mean values ± s.d.; sample size = 6). (**c**) Representative H&E, Masson’s Trichrome and immunofluorescence-stained sections obtained at the skin-tissue interface between the integrated patch and the skin wound site in each group (Scale bar = 250 μm).


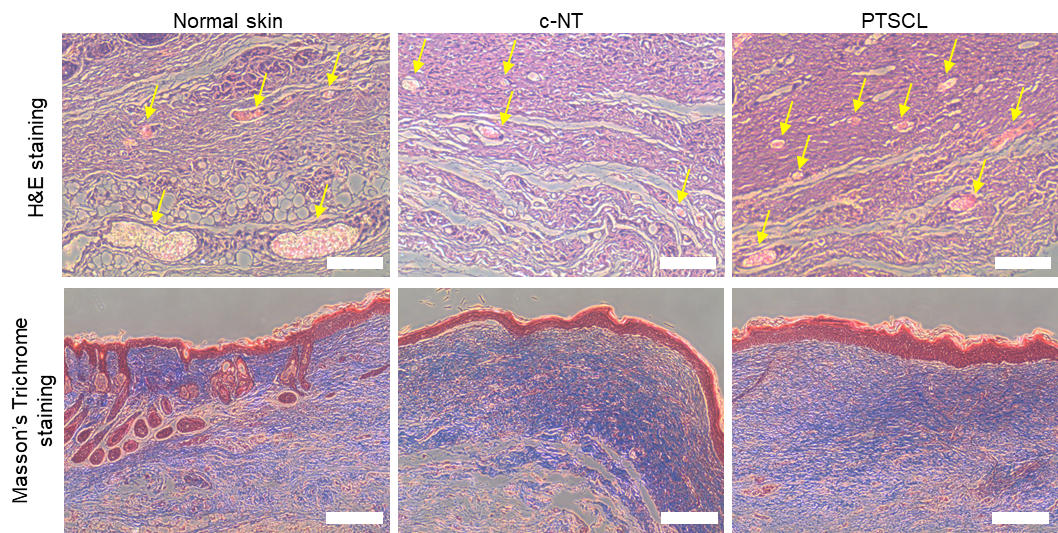


Figure S34. Hematoxylin and Eosin (H&E) staining and Masson’s Trichrome (MT) staining for skin histology after 28 days of wound formation. Yellow arrows in H&E staining indicate blood vessel (Scale bar = 125 μm for H&E staining, 250 μm for MT staining)


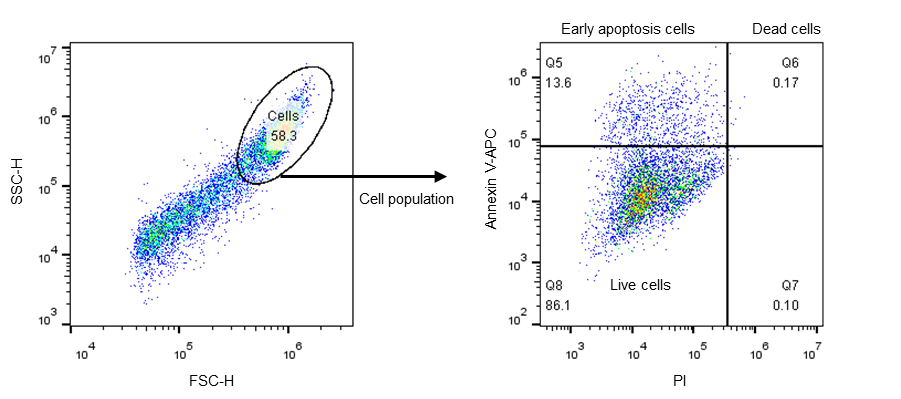


Figure S35. Gating strategy of flow cytometry.

**Table S1. Sequences for qRT-PCR.**

| **Gene** | **Forward primer (5′→3′)** | **Reverse primer (5′→3′)** |
| --- | --- | --- |
| *Human GAPDH* | GTCGGAGTCAACGGATTTGG | GGGTGGAATCAATTGGAACAT |
| *Human HSP27* | GGGCAACACCTCTACAAGGA | CGACTCGAAGGTGACTGGGA |
| *Human HSP70* | ATGTCGGTGGTGGGCATAGA | CACAGCGACGTAGCAGCTCT |
| *Human HSP90-α* | CTTGGGTCTGGGTTTCCTC | GGGCAACACCTCTACAAGGA |
| *Human Hif-1α* | CAGGTGACTGCTATCCCGTC | GTGCAGCGTGCAATACTAGC |
| *Human VEGF* | GAGGGCAGAATCATCACGAAG T | CACCAGGGTCTCGATTGGAT |
| *Human HGF* | GAT GGC CAG CCG AGG C | TCA GCC CAT GTT TTA ATT GCA |
| *Human FGF2* | GAC GGC AGA GTT GAC GG | CTC TCT CTT CTG CTT GAA GTT |
| *Human CXCR4* | TACACCGAGGAAATGGGCTCA | AGATGATGGAGTAGATGGTGGG |
| *Human CXCL12* | GAT TGT AGC CCG GCT GAA GA | GTG GGT CTA GCG GAA AGT CC |
| *Human MMP-2* | AAAATGGATCCTGGCTTCCC | AATAGGCGCCCTTGAAGAAGT |
| *Human PDGF* | GCACCGAGGTGTTCGAGAT | CTGCACGTTGCGGTTGTT |
| *Human KGF* | CGC AAA TGG ATA CTG ACA CG | GGG CTG GAA CAG TTC ACA CT |
| *Human Ki-67* | TGACCCTGATGAGAAAGCTCAA | CCCTGAGCAACACTGTCTTTT’ |
| *Human PCNA* | AGG GCT GAA GAT AAT GCT GAT ACC | CTC CTG TTC TGG GAT TCC AAG TTG |
| *Human TGF-β1* | CCCAGCATCTGCAAAGCTC | GTCAATGTACAGCTGCCGCA |
| *Human IL-4* | ACTTTGAACAGCCTCACAGAG | TTGGAGGCAGCAAAGATGTC |
| *Human BAX* | CATGTTTTCTGACGGCAACTTC | AGGGCCTTGAGCACCAGTTT’ |
| *Human BCL-2* | ATCGTCGCCTTCTTCGAGTT | ATCCCATCCTCCGTTGTCCT |
| *Human Caspase-3* | CGGCGCTCTGGTTTTCGTTA | CAGAGTCCATTGATTCGCTTCC |
| *Mouse IL-1* *β* | GCA ACT GTT CCT GAA CTC AAC T | ATC TTT TGG GGT CCG TCA ACT |
| *Mouse CD86* | CTTACGGAAGCACCCACGAT | CGGCAGATATGCAGTCCCAT |
| *Mouse STAT1* | GATCGCTTGCCCAACTCTTG | ACTGTGACATCCTTGGGCTG |
| *Mouse Arg1* | AAC ACG GCA GTG GCT TTA AC | GTC AGT CCC TGG CTT ATG GTT |
| *Mouse CD206* | AAA CAC AGA CTG ACC CTT CCC | GTT AGT GTA CCG CAC CCT CC |
| *Mouse STAT6* | GAGCTACTGGTCAGATCGGC | GGATGACGTGTGCAATGGTG |
| *Mouse Fibronectin* | GACCCCCTTCATCACCAACC | TGTCCGCCTAAAGCCATGTT |
| *Mouse Keratin 14* | CCG ACC TGG AGA TGC AGA TT | GCC ACC TCC TCG TGG TTC |
| *Mouse Involucrin* | CCTGTGAGTTTGTTTGGTCTACA | GAACCACAGCTGGAACAGTC |
| *Mouse CD31* | GTG GTG CTG ATG TCC ACA AG | AAC AGT GTC TGC CAT CCT TCT |

**Table S2. Comparison of wound healing outcomes across in vivo test groups.**

| Groups | Wound healing outcomes | | |
| --- | --- | --- | --- |
|  | Cell delivery | Skin maturation | Angiogenesis |
| Teg-NT | **×** | **×** | **×** |
| c-NT | **√** | **×** | **×** |
| c-LED | **√** | **√** | **×** |
| PTSCL | **√** | **√** | **√** |
| c-Heat | **√** | **×** | **√** |

**Abbreviation:** **Teg-NT:** No treatment, wounds covered with the commercial skin dressing Tegaderm; **c-NT:** Au/Nanoturf membrane-embedded skin wound patch + stem cell spheroid + Tegaderm ; **c-LED:** PDMS patch + stem cell spheroid + wireless LED module + Tegaderm; **PTSCL:** Photothermal-stem cell-light therapy (Au/Nanoturf membrane-embedded skin wound patch + stem cell spheroid wireless LED module + Tegaderm); **c-Heat:** Au/Nanoturf membrane-embedded skin wound patch + stem cell spheroid + wireless heater module + Tegaderm

l
